# Inhibition of NF-kB and COX-2 by andrographolide regulates the progression of cervical cancer by promoting PTEN expression and suppressing PI3K/AKT signalling pathway

**DOI:** 10.1038/s41598-024-57304-7

**Published:** 2024-05-26

**Authors:** Akbar Pasha, Kiran Kumar, S K Heena, I. Arnold Emerson, Smita C. Pawar

**Affiliations:** 1 Department of Genetics and Biotechnology, University College of Science, Osmania University, Hyderabad, Telangana 500007 India; 2 Department of Bioinformatics, School of Biosciences and Technology, Vellore Institute of Technology, Vellore, Tamil Nadu 632014 India; 3 Department of Pathology, Osmania Medical College, Hyderabad, Telangana 500095 India

**Keywords:** Andrographolide, Cervical cancer, NF-kB, COX-2, PI3K, PTEN, Cancer, Computational biology and bioinformatics

## Abstract

In the face of recent advances in Cervical cancer (CC) treatment, therapeutic and surgical procedures for CC management are still inadequate. In the current study for the first time Andrographolide (Andro) has been explored for its multitarget therapeutic efficacy on NF-kB, COX-2, and PI3K/AKT expressions together in CC. The expression levels of NF-kB, COX-2, PI3K and PTEN in the CC patient samples, both at mRNA and protein levels have shown significant association with poor survival and increased tumor aggressiveness. The binding efficacy of Andro was investigated using molecular docking and molecular dynamic simulations, and the protein and ligand complex for NF-kB and COX-2 has shown high binding energy. Andro displayed cytotoxicity by impeding the in-vitro proliferation of CC cells. Andro significantly supressed the NF-kB, COX-2, and PI3K expression and enhanced the expression levels of PTEN at protein levels in-vitro. Andro induced apoptosis in a dose dependent manner and significantly inhibited the migration and invasion of CC cells. Andro exhibited similar activity in-vivo and suppressed the CC tumor growth in xenograft C57BL/6 mice model. The anti-tumor activity of Andro, both in-vitro and in-vivo has shown considerable downregulation of NF-kB and COX-2 and induced apoptosis through impeding the PI3K/AKT signalling pathway. These findings from the above study projects, administration of Andro as an effective alternate safe compound to curtail and impede cervical cancer progression.

## Introduction

Cancer of the cervix uteri is the fourth most common cancer among women worldwide, with an estimated 604,127 (3.1%) new cases and 341,831 (3.4%) deaths. In India CC is the third most cancer with an estimated 123,907 (18.3%) cases and second leading cause of death with a mortality rate of 9.1%. as per GLOBOCAN 2020^1^. Poor prognosis due to late diagnosis and women with persistent infection of HPV-caused increase in incidence of CC and further worsened by limited treatment options. Other risk factors for CC include, socio economic status, traditional practises and beliefs, marriage at a young age, having multiple sexual partners, number of pregnancies, poor genital hygiene, lack of awareness, use of oral contraceptives, smoking, and HIV co-infections^2–4^. Surgery, radiotherapy, and hormone chemotherapy are currently in use therapeutic strategies^5,6^. Long-time effect of treatments for CC impacts the quality of life of CC survivor, whether treatment was surgical, radiotherapy or combination of both, they have greater persistent bladder, bowel, and sexual dysfunction after many years of treatment^7^. The conventional therapeutic and surgical procedures for CC management have been shown to be insufficient. Due to the difficulty of designing personalised treatments, drug resistance and dose-limiting toxicities are also impeding the therapeutic effect and prognosis of CC patients. Therefore, it is essential to devise some novel therapeutic strategy with effective agents with minimal side effects to improve patient’s quality of life. Plants are a valuable source of such promising molecules and plant based natural products has a very significant role in the fields of pharmacy, medicine and biology^8,9^.

Andrographolide (Andro), a diterpenoid lactone isolated from leaves of *Andrographis paniculata,* commonly used to treat diseases such as bacterial infections, diarrhoea, laryngitis, rheumatoid arthritis, anti-malaria and anti-inflammatory diseases in Southeast Asian countries^10–13^. Earlier studies has shown that Andro has antitumor activity in a variety of cancers such as melanoma cancer^12^, breast cancer^13^, colorectal cancer^14^. In Alzheimer's disease, Andro effectively activates nuclear factor like2 (Nrf2) mediated heme oxygenase-1 expression while inhibiting Aβ42 (amyloid beta) overexpressed microglial BV-2 cell activation^15^. Andro inhibits in-vitro angiogenesis by inhibiting MMP-2 and MMP-9, and by regulating transcription factor NF-kB nuclear translocation in human umbilical vein endothelial cells^16^. Transcription factor NF-kB plays a key role in inflammatory responses and cancer development by regulating a number of genes involved in angiogenesis and tumorigenesis^16,17^. There are five related NF-kB proteins: p50 (NF-kB1), p52 (NF-kB2), p65 (RelA), RelB, and cRel^18,19^. IKK and the IkB family of proteins are upstream signalling molecules for the NF-kB pathway^20^. By binding to its inhibitor IkB, NF‐kB—is kept inactive within the cytosol^21^. Extracellular stimuli IL-1β dissociates NF-kB from cytoplasmic inhibitors, resulting in translocation of NF-kB p65 to the nucleus and binding to specific DNA sequences^22^. Several potential transcriptional regulatory elements have been identified in the human COX-2 gene, including a peroxisome proliferator response element (PPRE), two cyclic AMP response elements (CRE), a sterol response element (SRE), two nuclear factor kappa B (NF-kB) sites, specificity protein1 (SP1) site, a nuclear factor for interleukin-6 expression (NF-IL6) motif and a TATA box^23^. The human COX-2 gene promoter region contains two motifs that are similar to the NF-kB consensus binding site. The presence of p50/p65 in the nucleus, as well as further binding of p65 at the COX-2 promoter region, most likely contributes to COX-2 expression^24–26^.

Prostaglandins (PG) are synthesised from arachidonic acid by cyclooxygenases (COX). COX is divided into two isoforms, COX-1 and COX-2 while COX-1 is constitutively expressed in most tissues and appears to be involved in housekeeping functions, on the other hand, COX-2 is not found in most normal tissues but is activated by cytokines, growth factors, carcinogens, and tumor promoters^27–29^. However, COX-2 is commonly over expressed in the majority of cancers, including adenocarcinoma, squamous cell carcinoma (SCC), transitional cell carcinoma, endometrial carcinoma, cholangiocarcinoma, and hepatocellular carcinoma^24,30–32^. Tumor microenvironment (TME) is an inducer for COX-2 overexpression and this is due to dysregulation of its transcriptional or post-transcriptional levels. Overexpression of COX-2 promotes carcinogenesis and increase the rate of cancer recurrence and reduces the survival of patients and it is related to cancer cell resistance to chemotherapy and radiotherapy^33–35^.

COX2 plays a crucial role in the activation of the PI3K/AKT pathway in cancer cells by suppressing PTEN (phosphatase and tensin homologue)-an essential tumor suppressor factor, either directly^36^ or indirectly through suppression of TET1induced PTEN activation^37^. The PI3K/AKT/mTOR (mammalian target of rapamycin) signalling pathway regulates cell growth, survival, motility, metabolism, and angiogenesis, and its activation contributes to tumor development and resistance to anticancer therapy^38^. PTEN is a major negative regulator of the PI3K/AKT signalling pathway that promotes cell survival and inhibits apoptosis by dephosphorylating PIP3 (Phosphatidylinositol (3,4,5)-trisphosphate) to PIP2 (Phosphatidylinositol (4,5)-bisphosphate)^39,40^. Therefore loss of PTEN is a common cause of PI3K activation and knockdown of PTEN increase cell spreading, migration, invasion and tumor size in nude mice^41^.

Previously it has been reported that Andro causes cell cycle arrest of human colorectal carcinoma^42^, and also reduces inflammation by inhibiting NF-kB activation via covalent conjugation of p50 reduced cysteine62^43^. Further, Andro promotes NF-kB subunit p65 Ser-536 dephosphorylation in vascular smooth muscle cells by activating protein phosphatase2A^44^. Andro inhibited the growth and spread of luminal-like breast cancer through the downregulation of NF-kB and miRNA-21-5p^45^. Andro inhibits breast tumor growth in an orthotopic NOD/SCID mouse model and the inhibitory effect of Andro on pro-angiogenic molecules such as OPN and VEGF expression associated with decreased PI3K/AKT activation^46^. Other studies have shown that Andro can inhibit COX-2 expression by decreasing p300 HAT activity, which reduces NF-kB acetylation and suppresses angiogenesis via the VEGF pathway^47^. In our previous study, we found that Andro has an anti-inflammatory effect by inhibiting iNOS, thus reducing NO production and tumor cell proliferation, migration, and induced apoptosis, cell cycle arrest from G1 to S phase in human cervical HeLa cell lines^48^. However, the inhibitory effect of Andro on the inflammatory and survival pathways in cervical squamous cell carcinoma are yet to be elucidated. Targeting multiple inflammatory markers, such as NF-kB and COX-2, which regulate the PI3K/AKT/PTEN pathway and cell proliferation is a novel approach for treating cervical cancer.

In present study we investigated the expression levels of NF-kB, COX-2, PI3K and PTEN both at mRNA and protein level in CC patients. Molecular docking and molecular dynamic simulations were used to investigate Andro's binding efficiency and molecular interactions with NF-kB and COX-2. Our in-vitro findings indicate that Andro inhibited cervical cancer (HeLa and SiHa cells) cell proliferation, migration and invasion. Andro inhibits the expression of inflammatory molecules such as NF-kB and COX-2, can significantly increase PTEN expression and impede the activation of PI3K/AKT pathway in-vitro and in-vivo. In xenograft of CC model, intraperitoneal administration of Andro reduced tumor weight and volume. All together our findings suggest that Andro could be used as a potential therapeutic agent in the treatment of cervical cancer.

## Materials and methods

### Reagents, antibodies and cell lines

Andrographolide (Andro) was obtained from Sigma Aldrich (St. Louis, MO, USA, Product number 365645; Lot # MKCF4812) and prepared as a 100 mM stock solution in DMSO (dimethylsulphoxide), further diluted with PBS. MTT (3-(4, 5-dimethylthiazole-2-yl)-2, 5-biphenyl tetrazolium bromide) (Promega, Madison, Wisconsin, USA). Matrigel (BD Biosciences, San Diego, CA, USA), transwell chambers (8 μm, Gyeonggi-do, Korea, genetix Biotech Asia Pvt. Ltd.). DMEM (Dulbecco's Modified Eagle Medium) with high glucose (#AL007S), foetal bovine serum (#RM9955) and trypsin were obtained from Himedia Laboratories (India). Primary antibodies, anti-Bcl-2 (MA5-11757) and anti-Bax (MA5-14003) obtained from Invitrogen. Anti-NF-kB p-65 (mAb#3036) anti-COX-2 (SC-19999), anti-PI3K p-85α (SC-1637), anti-GAPDH (SC-25778), anti-actin (SC-4778) and all the secondary antibodies obtained from Santa Cruz Biotechnology (Santa Cruz, California, USA.). Anti-AKT (#4685), anti-p-AKT (#4060), procured from Cell Signaling Technology (Cell Signaling Technology, Inc., USA). Human cervical cancer cell lines (HeLa and SiHa), human embryonic kidney (HEK-293) cells were obtained from the National Centre for Cell Science (NCCS) Pune, India and were grown in Dulbecco Modified Eagle's Medium (DMEM) supplemented with 10% FBS and 1% penicillin and streptomycin antibiotics. The cells were incubated at 37° C in a 5% CO_2_ incubator.

### Investigation of cervical tissue biopsy samples

#### Tissue biopsy collection

A total of 60 female subjects were recruited for the study, which included 40 cervical squamous cell carcinoma tissue biopsies and 20 normal cervical tissue biopsies. The invasive CC tissue biopsy specimens (n = 40) were obtained from the oncology unit at Mehdi Nawaz Jung Hospital (Hyderabad, India). None of the patients received preoperative chemotherapy or radiation therapy. Healthy cervical tissue specimens (n = 20) were collected from women with no prior history of cancer, or cervical intraepithelial neoplasia, and with normal cervical cytology, and who had underwent hysterectomy at the Department of Gynaecology, CC Shroff Hospital, Hyderabad, India. A portion of the biopsy sample was excised and fixed in formalin for histopathological analysis, and the remaining tissue specimens were collected in RNA*later* (Sigma #R0901) and stored at − 80 degrees Celsius prior to utilization. The Institutional Ethical Committee of Osmania University and MNJ Cancer Hospital approved the study for biomedical research (Regd. No: ECR/227/Inst/AP/2013/RR-19). Informed consent was obtained from all subjects and /or their legal guardian(s) involved in the study.

#### Immunohistochemistry (IHC)

Immunohistochemistry was performed on formalin-fixed, paraffin-embedded biopsy samples from cervical cancer, normal cervix tissues, and mice tumor tissues were sectioned to 4-µm thickness with a microtome and mounted on poly-L-lysine-coated glass slides, as previously described^49^. Briefly; Paraffin slides were deparaffinised by baking at 55 °C for 60 min, then soaked in xylene and passed through graded alcohols for rehydration. Antigen retrieval was accomplished by incubating tissue sections in 0.01 M citrate buffer (pH 6.0–6.2) for 15 min. H_2_O_2_ (3% in methanol) was used to block endogenous peroxidase activity for 20 min. The slides were incubated with 10% goat serum for 10 min to reduce nonspecific antibody binding. The sections were incubated with a 1:100 dilutions of anti-PI3K, anti-NF-kB, anti-COX-2, and anti-PTEN specific monoclonal antibodies for 1 h. The sections were then incubated with biotin-labeled secondary antibody anti-mouse IgG for 15 min followed by treatment with streptavidin peroxidase reagent for 10 min. The specimens were washed with PBS solution followed by incubation with DAB (diaminobenzidine) chromogen as per manufacture’s protocol and counterstained with hematoxylin. The section slides were dehydrated with ethyl alcohol and Xylene followed by mounting with DPX solution. The specimen sections were then examined and photographed using an inverted light microscope (Magnüs INVI Olympus, Noida, India) at 40X magnification. The staining intensity of the specimen was graded from 0 to 3, with 0 indicating negative staining (0–5%), 1 indicating weak staining (6–25%), 2 indicating moderate staining (26–50%), and 3 indicating strong staining (51–100%)^50,51^. This staining intensity was used to quantify the altered gene expression score.

#### RNA extraction and quantitative RT-PCR analysis

Total RNA was extracted from cervical squamous cell carcinoma and normal cervix tissue biopsies using the Qiagen universal kit (Cat No: 73404) according to the manufacturer's protocol. The RNA quality and quantity was evaluated using an Eppendorf nanodrop spectrophotometer and cross-validated using 1.2% agarose gel electrophoresis (SeaKem®LE Agarose Catalog: #50,004). A total of 1 µg of RNA was reverse-transcribed from both tumor and non-tumor samples using iScript™ cDNA synthesis kit (Biorad, Cat#1,708,891), and 1 µl of reverse-transcribed RNA was used to determine gene expression using SYBR Green Master Mix (KAPA SYBR® FAST (2X) Universal, Cat no: KK4601) in Agilent Ariamx real-time PCR detection system with a total reaction volume of 20 µl in each well. Gene primers and amplicon size are depicted in Table 1. Each reaction was performed in triplicate, and the beta-actin gene was used as a reference endogenous control. Endogenous control was used to normalise the mRNA level in each sample. The Livac method 2^− ΔΔct^ was used to calculate relative gene expression^52^. GraphPad Prism 6.01 (GraphPad Software Inc., San Diego, CA, https://www.graphpad.com) was used for statistical analysis of qPCR, and the mean ± standard error (SME) of all values was calculated using the student t-test with a significant p value of < 0.05.

**Table 1:** Gene name, primer sequences, and amplicon size used in RT-qPCR.

Gene	Forward primer	Reverse primer	Product length(bp)
PI3K	GGAGCCCAAGAATGCACAAA	GCATTCCAGAGCCAAGCATC	133
NF-kB	ATTGCGGACATGGACTTCTC	CCCACCAGAATCCGTAAGTG	128
COX-2	CGCTCAGCCATACAGCAAAT	ATTCCGGTGTTGAGCAGTTT	138
PTEN	TGCCATCACCATTTCCATTGA	CCAAAGAGTTTCATGTTAGCAGC	126
ACTIN	CACCATTGGCAATGAGCGGTTC	AGGTCTTTGCGGATGTCCACGT	135

#### Receiver operating characteristic (ROC) curves

The ROC curve is a graphical technique used to assess a test's ability to differentiate between disease and non-disease individuals (cancer and non-cancer). The ∆CT values of each sample were used to create a receiver operating characteristic (ROC) curve. The curve is created by calculating the test's sensitivity and specificity at each possible cut-off point and plotting sensitivity on the y axis versus 1-specificity on the x axis. The area under the curve (AUC) value defines the usefulness of markers in terms of their ability to differentiate between two groups of cervical tissue biopsies (patients with and without cancer). The ROC curve was used to calculate the highest Youden (Y) index, and the Y-index is associated with the ideal cut-off point (COP). The COP values indicate increased or decreased gene expression levels in cancer and normal samples. All of the plots were generated by using R-Studio version 3.6.3, htts://www.R-project.org/^53^.

### In silico analysis

#### Molecular docking

- (1) Preparation of ligand

Molecular docking was performed to identify the binding activity of the ligand-based on the binding affinity score. In this study, we have considered the natural compound Andro as a ligand relying upon its biological activity. The 3D structure of the phytochemical Andro in SDF format was downloaded from PubChem https://pubchem.ncbi.nlm.nih.gov/compound/5318517. Energy minimization was carried out and obtained the ligand pdbqt file with the least confirmation E = 535.66. Later AutoDockVina was used to perform docking to explore the best binding activity.

- (2) Preparation of the proteins

The 3D structures of protein targets Cyclooxygenase-2 (COX-2) and I-Kappa-B-Alpha/NF-Kappa-B complex (NF-kB) were downloaded in PDB format from Protein Data Bank with protein ID: 5IKT and 1NFI (https://www.rcsb.org/structure/5IKT & https://www.rcsb.org/structure/1NFI) respectively. Random coil for the proteins 1NFI chain A with 301 residues and protein 5IKT chain A with 552 residues was predicted using (https://npsa-prabi.ibcp.fr/cgi-bin/npsa_automat.pl?page=/NPSA/npsa_sopma.html). 1NFI, 155 out of 301 residues (i.e., 51.50%) contribute to the random coiling, and 264 out of 552 residues (i.e., 47.91%) aggregates for random coiling in 5IKT. Proteins 1NFI and 5IKT were loaded onto PyRx and converted to macromolecules by adding Kollman charges with due converting the proteins to pdbqt format. Water was removed and polar hydrogens were added.

- (3) Ligand and proteins docking

Further blind docking was carried out by a selection of ligand and macromolecules followed by grid selection. The grid box for 1NFI and 5IKT was set respectively. Both the proteins 1NFI and 5IKT were docked by using AutoDockVina in PyRx-GUI, where the binding affinity of the ligand to the proteins was determined as − 7.2 kcal/mol for 1NFI and − 8.0 kcal/mol 5IKT.

#### Molecular dynamic simulation (MDS)

Molecular dynamic simulation is used to investigate the dynamics of the molecules at the atomic level. In this study, MD simulation for prepared proteins 1NFI (I-Kappa-B-Alpha/NF-Kappa-B complex) and 5IKT (Cyclooxygenase-2 (COX-2)) in interaction with ligand Andro interactions and their dynamics were studied using GROMACS 2020.2 package GROMOS96 43a1 force field. The topological parameters were determined for the ligand—Andro using the PRODRG server. Four MDS runs were carried for two proteins where the native structure of each protein and two complexes with Andro. These protein complexes were placed in the unit cubic box at a distance of 1.0 nm from the molecule to the edge. This unit cubic box around the molecule was solvated with SPC—(simple point charge) water model. The total charge of the system was neutralized by Cl^−^ ions, followed by energy minimization of 50,000 steps with convergence tolerance of 1000 kJ/mol nm^−1^. To proceed with MD, the system should be equilibrated under NVT (constant particles, volume, and temperature) and NPT (constant particles, pressure, and temperature) for about 100 ps. Finally, MD simulation was performed for a 100 ns timescale.

The first phase of molecular dynamics was employed to investigate the conformational stability of the native protein structure of 1NFI and its complex structure with ligand Andro. In the second phase native structure of 5IKT and its complex structure with ligand, Andro was engaged to evaluate the conformational stability in association with both 1NFI and 5IKT. The RMSD, (Root mean square deviation), RMSF (Root mean square fluctuations), Hb (Hydrogen bonds), Rg (Radius of gyration), SASA (Solvent accessible surface areas), and Protein–Ligand interaction energy plot for native and complex structures of proteins (1NFI and 5IKT) were chosen as parameters to analyse MD trajectory using GROMACS utilities and the results were plotted using Xmgrace.

### In-vitro analysis

#### Cell viability/MTT assay

The cell viability assay was performed as previously described^48^. In brief, 3 × 10^3^ cells/well were seeded into 96-well culture plates and incubated at 37 °C in 5% CO_2_ for 24 h. Cells were treated with or without Andro at increasing concentrations (0, 5, 10, 20, 40, and 80 µM) for 24 h in a final volume of 200 µl of fresh DMEM medium. After 24 h, 20 µl of MTT (5 mg/ml in PBS) was added to each well and incubated at 37 °C for 4 h. Following that, the media was removed and the plates were incubated in a shaker incubator for 30 min with 100 µl of DMSO (Dimethyl sulfoxide). The absorbance was measured at 595 nm using Multiskan FC micro plate reader (Thermo scientific). Three independent experiments were performed. Cell viability was calculated for each treated cells using untreated cells as control. The inhibitory concentration (IC_50_) was calculated using Graph-pad Prism software.

#### Transwell migration assay

Cell migration assay was performed using 24 well transwell chambers. HeLa and SiHa cells were seeded at a density of 5 × 10^4^ cells (300 µl/well) in the upper chamber using serum-free media, while the lower chamber was filled with 700 µl of DMEM medium supplemented with 12% FBS as a chemo attractant. The cells were treated with 5 and 10 µM Andro and incubated for 16 h at 37 °C followed by fixed with 4% formaldehyde. The cells were washed twice with PBS and stained with 10% crystal violet. The non-migrant cells were removed from the upper surface of the chamber by using cotton swabs. The inhibition of cell migration by Andro was assessed by counting the cells using ImageJ software (version 1.50i, National Institutes of Health, Bethesda, MD, USA) from three different images captured with a magnification of 20X using an inverted microscope (Magnüs INVI Olympus, Noida, India).

#### Transwell invasion assay

The motility of human cervical cells (Hela and SiHa) was performed in a 24 well transwell chamber. Matrigel was obtained from BD Biosciences and diluted in serum free medium, 50 µl of the diluted matrigel was inoculated into the upper chamber to form a gel at 37 °C for 30 min. Then 5 × 10^4^ cells (Hela and SiHa) per well were seeded in the upper chamber in 300 µl of serum free media and the lower chamber was filled with 700 µl of complete medium supplemented with 12% FBS as chemoattractant. Further the cells were treated with Andro (0, 5 and 10 µM) and incubated for 16 h at 37 °C followed by wells were fixed with 4% formaldehyde. The cells were stained with 10% crystal violet for 15 min at room temperature, then rinsed twice in PBS. The non-invasive cells from the upper membrane surfaces were removed by wiping with cotton swabs. The invasive cells were photographed at a 20 × magnification under an inverted microscope (Magnüs INVI Olympus, Noida, India). Cells were counted using ImageJ software (version 1.50i, National Institutes of Health, Bethesda, MD, USA, https://imagej.net/ij/).

#### Western blot analysis

Western blot analysis was carried out as previously mentioned^48^. Briefly Hela and SiHa cells were treated with Andro with different concentrations (0, 2, 4, 8, and 10 µM) for 24 h. Total protein was extracted by the cells were resuspended and lysed for 1 h at 4 °C in RIPA (radioimmunoprecipitation assay) buffer (Cat. No. R0278). The cells were centrifuged at 12,000 rpm for 30 min at 4 °C and the Bradford assay was used to estimate the protein concentration. An equal amount of the 20 µg protein lysate from each sample was resolved by SDS-PAGE which was then blotted onto a nitrocellulose membrane (0.2 µM Bio-Rad, cat. no. 1620112). The membrane blocked with 5% skimmed non-fat dry milk and incubated with anti-Bcl-2 (1:2000), anti-Bax (1:2000), anti-NF-kB p-65 (1:2000), anti-COX-2 (1:1000), anti-PTEN (1:3000), anti-PI3K p-85α (1:1000), anti-AKT (1:2000), anti-p-AKT (1:2000), anti-GAPDH (1:2000) and ant-Actin (1:1000) antibodies overnight at 4 °C. Then after the membranes were rinsed three times with TBS (Tris buffered saline) for ten minutes each followed by incubation with HRP-conjugated secondary anti-mouse or anti-rabbit antibodies (1:5000 dilutions) for 1 h at room temperature and then washed thrice with TBST (Tris buffered saline containing tween-20, pH-7.5). The immunoblots were visualised using ECL-substrate (Clarity™ western ECL substrate Cat# 170-5061) under the ChemiDoc MP Imaging System (Bio-Rad). All the experiments were carried out in triplicates.

### Animal studies

Female mice C57 BL/6, 4–6 weeks old, were purchased from animal breeding facility of Vyas labs, Hyderabad, India (CPCSEA Reg. No: 2085/PO/RcBiBt/S/19/CPCSEA). All the experimental procedures and protocols were reviewed and approved (OU-IAEC-001/GEN/2021) by the Institutional Animal Ethics Committee (IAEC) of the Department of Genetics, Osmania University, prior to the initiation of the experiment and the study was performed in accordance with relevant guidelines and regulations. The study is reported in accordance with ARRIVE (Animal Research: Reporting of In-Vivo Experiments) guidelines. The mice were kept in pathogen-free environments and maintained at 25 ± 2 °C with a 12-h cycle of light and darkness and they had free access to food and water ad libitum. Prior to the tumor induction the mice were immunosuppressed with 0.5 mg/kg of Dexamethasone, was injected for 7 days. A total of 1 × 10^7^ HeLa cells were inoculated subcutaneously in 200 µl of serum free medium into the right flank of each mouse. Following inoculation, the tumor development was carefully monitored, and starting on day 3, the tumor volume was assessed using digital Vernier caliper. When the tumor volume reached ∼200 mm^3^, the mice were randomly divided into 3 groups, control group, treatment group-I and treatment group-II and each group contained four mice (n = 4). Mice in the treatment group-I & II were intraperitoneally injected with 100 µl of Andro (twice a week) at a dose of 15 mg/kg and 30 mg/kg per body weight and the control group was treated with an equal volume (100 µl) of saline. The mice body weight and the tumor volume were assessed every three days and the tumor size was calculated by standard formula: tumor volume = Length × Width^2^ × 1/2. All the experimental mice were sacrificed after 30 days and the tumors from each mouse were excised and their weights were recorded. To assess the expression of the indicated proteins, the tumor tissue was fixed in 10% formalin, embedded in paraffin and sectioned into 4 µm slices for further IHC analysis. A portion of the mice tumor tissue were collected in RNA*later* (Sigma #R0901) and stored at − 20 °C for subsequent western blot analysis.

### Statistical analysis

The data were obtained from three independent experiments and provided as the mean ± standard deviation (SD) for all experimental evaluations. The student’s t-test was used to evaluate the statistical comparison of the mean values between two groups. One-way ANOVA followed by Tukey's test was employed to assess the statistical differences between multiple groups. P value < 0.05 was considered to be statistically significant. All the statistical analyses were carried out using GraphPad Prism 6.01 (GraphPad Software Inc., San Diego, CA, https://www.graphpad.com).

### Ethical approval and consent to participate

The study was approved by the institutional ethical committee of Osmania University and MNJ Cancer Hospital (Regd. No: ECR/227/Inst/AP/2013/RR-19). All the patients and control subjects involved in the study given written consent. Animal experiments were approved by the Department of Genetics' Institutional Animal Ethics Committee (OU-IAEC-001/GEN/2021).

### Consent for publication

All authors reviewed the results and approved the final version of manuscript.

## Results

### Expression of NF-kB/COX-2 and PI3K/PTEN in cervical cancer tissue biopsies

**Figure 1:**
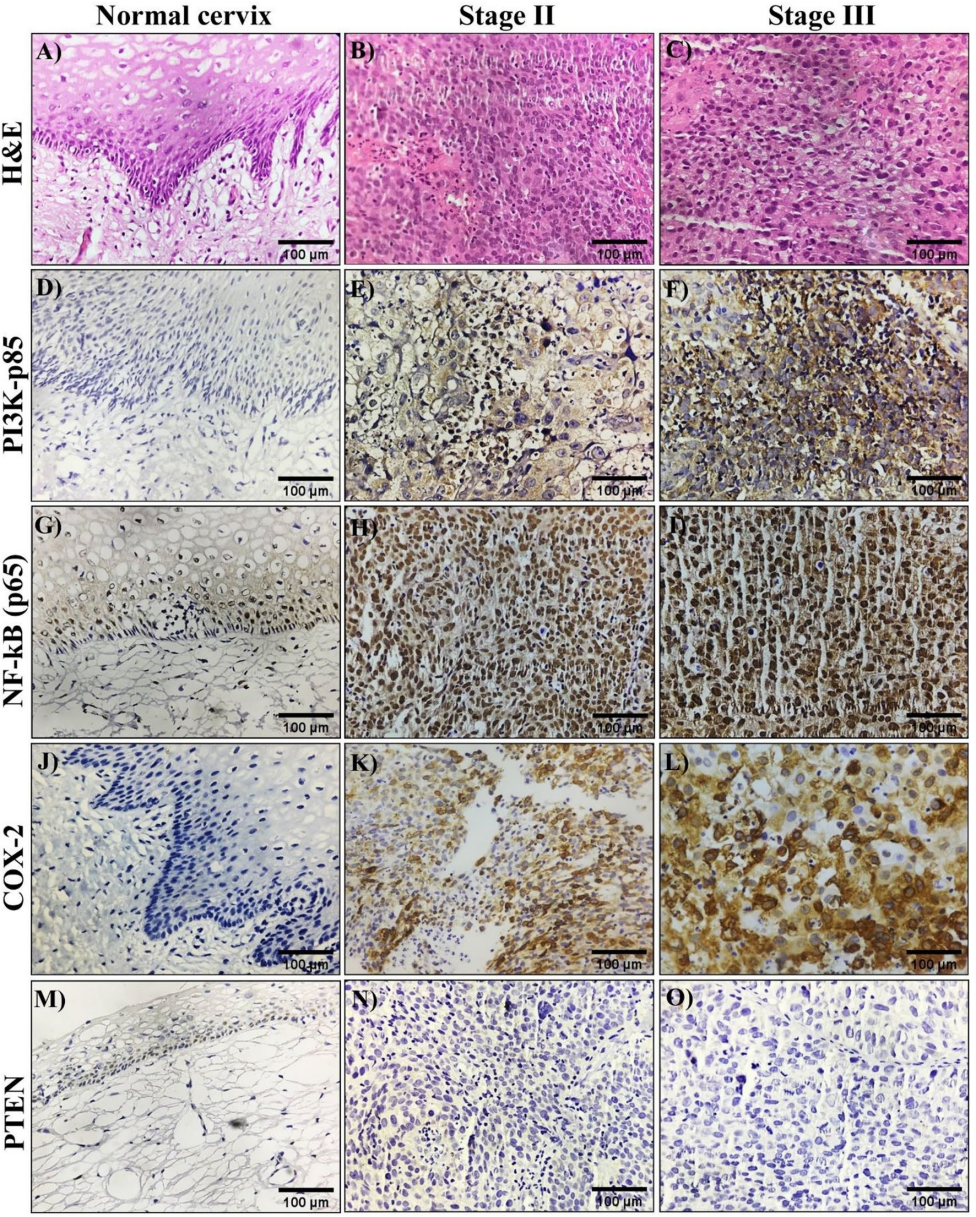
H&E and Immunohistochemical analysis of normal cervix and cervical cancer tissue biopsies. 40X magnification images of H&E staining of initially diagnosed cervical cancer tissues (**A**–**C**). H&E staining of Normal Cervix is lined by stratified squamous epithelium (**A**), stage-II micrograph showing atypical tumor cells (**B**), stage-III micrograph showing sheets and cords of tumor cells with nuclei are pleomorphic, hyperchromatic (**C**). PI3K, NF-kB-p65, COX-2, and PTEN immunohistochemical localization in normal cervix and cervical cancer tissue biopsies and brown staining signifies the presence of immunoreactive protein (**D**–**O**). Normal cervix tissue (**D**,**G**,**J**,**M**), stage-II cervical cancer tissue biopsies (**E**,**H**,**K**,**N**), and stage-III cervical cancer tissue biopsies (**F**,**I**,**L**,**O**).

Hematoxylin and Eosin (H&E) staining was used to investigate tissue samples from normal cervical tissue and patients tissue biopsies to identify the pathological morphology. The normal cervix is lined by stratified squamous epithelium and sub epithelium displaying fibro collagenous tissue (Fig. 1A). Stage II cancer cells were slightly aberrant and moderately differentiated, while stage III cancer cells were more abnormal and the tumours were defined as poorly differentiated. Stage-II micrographs (Fig. 1B) showed atypical tumour cells with mild to moderate pleomorphism, a vesicular nucleus, and associated inflammation, whereas stage-III cancer patients revealed sheets and cords of tumour cells, visible cytoplasm, Pleomorphic, hyperchromatic, and bizarre nuclei (Fig. 1C), indicating that cervical carcinoma tissue and cellular nuclei were damaged more as cancer progressed from stage II to stage III.

The immunoreactivity of NF-kB, COX-2, PI3K and PTEN was analysed using respective primary antibodies via IHC staining in cervical tissues. Results demonstrate the immunopositivity of PI3K as uniformly dark brown cytoplasmic and few nuclear staining, indicating substantially stronger expression in cervical malignant tissues with respect to cancer stage. Using the intensity of the grading, stage II showed dispersed moderate cytoplasmic positivity and few nuclei were stained (Fig. 1E), while stage III showed stronger expression in cytoplasm as well as nucleus was stained significantly (Fig. 1F). In normal cervical tissues, there was no discernible PI3K staining (Fig. 1D), and the results were negative. These findings revealed that, the PI3K levels in the CC tissues gradually increased as the tumor advanced from stage II to stage III.

The levels of NF-kB-p65 were positively expressed in the cytoplasm and nucleus of the cells in the CC cases, as opposed to basal NF-kB-p65 expression in the control cases (Fig. 1G). But in cases of stage-II, strong staining was mainly confined to the nucleus, and moderate cytoplasmic expression was seen (Fig. 1H). Further the expression of NF-kB-p65 is substantially stronger in stage-III cervical tissue samples, and the intensity of strong positive for the nucleus and cytoplasm increased with stage of cancer (Fig. 1I).

The IHC assay showed that COX-2 was expressed positively in the cervical squamous tissue samples, but not in the normal cervical tissue (Fig. 1J). COX-2 expression in tumor cells was primarily found in the cytoplasm, showing a diffuse distribution. The immunoexpression of COX-2 in stage III cervical tissue was found to be significant strong cytoplasmic positivity compared to moderate in stage II (Fig. 1K,L). Further, PTEN levels have been observed in normal tissues but not in cervical squamous tissue samples (Fig. 1M). However, immunohistological analyses demonstrated that PTEN levels in cervical cancer tissues decreased significantly as the tumor advanced from stage II to stage III (Fig. 1N,O).

### Gene expression analysis of NF-kB/COX-2 and PI3K/PTEN in cervical cancer tissue biopsies

**Figure 2:**
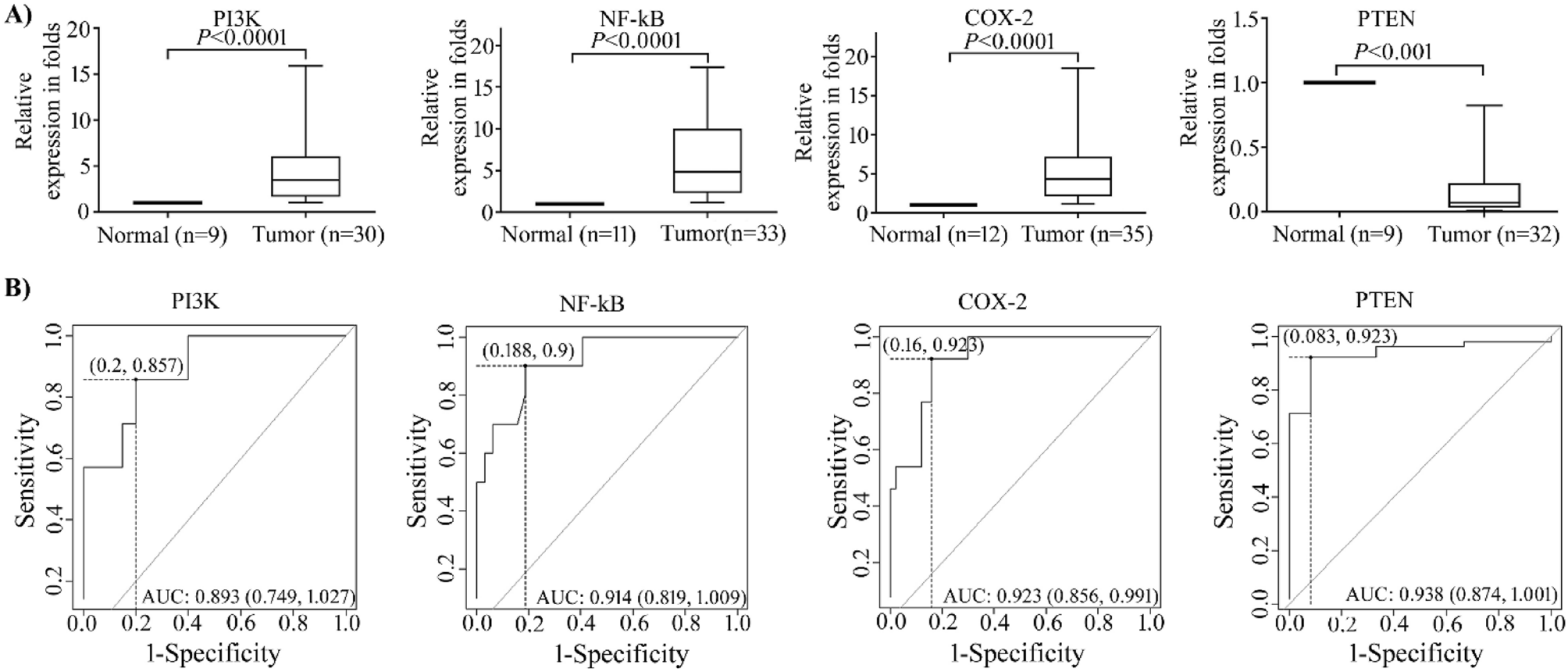
Relative gene expression and ROC (Receiver Operating Characteristic) curves: (**A**) Relative gene expression of NF-kB, COX-2, PI3K and PTEN was quantified by qRT-PCR in Cervical cancer tissue biopsy samples. Each individual experiment was normalized by housekeeping gene β-actin and relative expression was represented by Livac method (2^^-∆∆ct^). Data represent the Mean ± SEM (Standard Error of the Mean). (**B**) ROC curve based on RT-qPCR data for NF-kB, COX-2, PI3K and PTEN. The graph demonstrations a plot of true positive rate (Sensitivity) vs. false positive rate (1-specificity). The AUC values indicate that expression analysis of these markers may allow for the separation of the two groups. The highest Youden (Y) indices related to the COP are displayed at the point on the dotted line. The AUC (Area under the curve), Y (Youden index), COP (Cut-off point) and CI (Confidence interval) determined values were given in Table 2.

We analysed gene expression levels of NF-kB, COX-2, PI3K and PTEN from the CC group and control groups, the expression levels of NF-kB, COX-2, and PI3K were significantly overexpressed in all the cervical tissue biopsies while the expression of PTEN was noticeably decreased in all the cervical cancer tissue samples (Fig. 2A), housekeeping gene β-actin was used for the normalization gene expression. Box-whisker plots were clearly documented that the expression of inflammatory mediators NF-kB, COX-2, and survival mediator PI3K considerably increased in cervical cancer patients as compared to healthy controls, with a fold change 6.718 ± 0.9967, 5.722 ± 0.946 and 4.471 ± 0.8995 (Mean ± S.E) respectively. Subsequently PTEN expression was downregulated in CC patients compared to healthy controls with the fold change 0.1622 ± 0.0421 (Mean ± S.E) (Fig. 2A).

### Assessing the optimal cut-off point (COP) and diagnostic accuracy of NF-kB/COX-2 and PI3K/PTEN in cervical squamous carcinoma

Our data revealed that all the markers yielded a significant AUC value. As shown in Fig. 2B the overexpressed PI3K, NF-kB, and COX-2 obtained an AUC of 0.893, 0.914 and 0.923, respectively. The AUC value of the downregulated PTEN was 0.938. As a result, this analysis revealed that all four markers had a significant diagnostic value for differentiating CC patients from healthy controls (Fig. 2B Table 2). The highest Youden indices were 0.6571 for PI3K, 0.712 for NF-kB, 0.763 for COX-2, and 0.839 for PTEN respectively. The Youden index was associated to cut-off points (COPs) represented in ∆CT standards for differentiating the patients from healthy controls were 7.8 for PI3K, 8.35 for NF-kB, 13.42 for COX-2, and 3.73 for PTEN. A ∆CT value for the overexpressed genes PI3K, NF-kB, and COX-2 under the COP was regarded as positive for malignancy in accordance with an increase in these markers in cervical tissue biopsies, whereas the low expressed gene for PTEN, a ∆CT value over the COP was regarded as positive for cervical squamous carcinoma (Fig. 2B, Table 2).

**Table 2:** Statistical analysis of the cervical cancer patients versus healthy control group based on their RT-qPCR values.

Gene	AUC	Y-Index	COP (∆CT)	P-value	95% confidence interval
PI3K	0.893	0.6571	7.8	0.0023	0.759–1.027
NF-kB	0.914	0.712	8.35	0.0001	0.819–1.009
COX2	0.923	0.763	13.42	0.0001	0.856–0.991
PTEN	0.938	0.839	3.73	0.0001	0.874–1.001

### Andro interacts with NF-kB and COX-2

**Figure 3:**
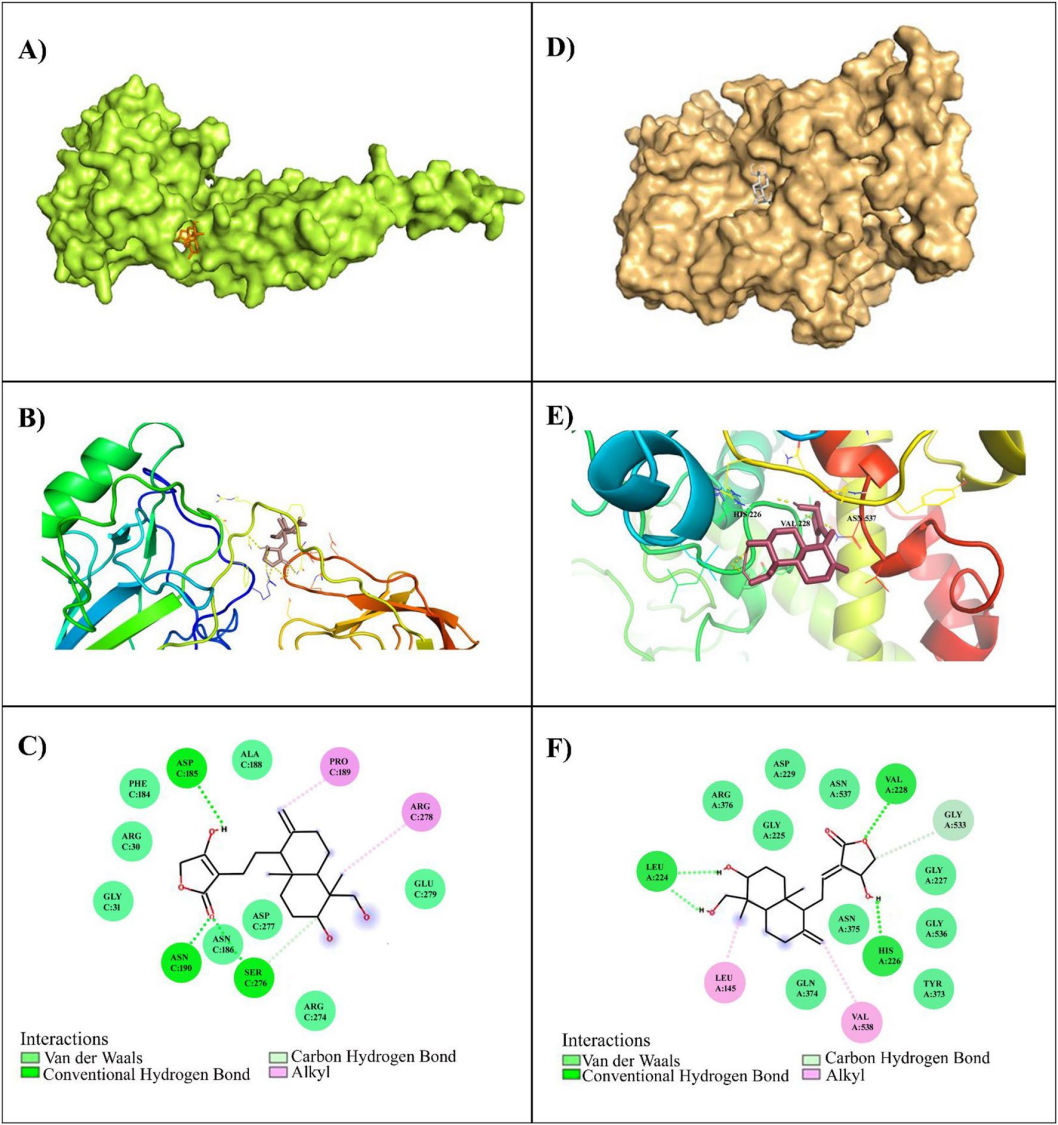
3D docking image of Andro with NF-kB (**A**), and COX-2 (**D**) (surface view). 3D ribbon representation of the binding cavity and interacting residues of NF-kB (**B**) and COX-2 (**E**) with Andro. Two-dimensional depiction of the non-covalent interaction between Andro and the catalytic active site of NF-kB (**C**), and COX-2 (**F**) and the inset shows an illustration in various colours of residues involved in interactions with van der Waals, hydrogen bonds, carbon-hydrogen bonds and alkyl.

Molecular docking studies were performed to explain the possible interaction between the natural compound, Andro and inflammatory receptor molecules NF-kB and COX-2 (Fig. 3A,D). The binding cavity and interaction residues of NF-kB and COX-2 with Andro are depicted in 3D ribbon form (Fig. 3B,E). The binding energy of the protein, NF-kB (1NFI) and the ligand, Andro was found to be − 7.2 kcal/mol. The results demonstrate the formation of non-covalent bonds including van der waals, hydrogen bonds, carbon-hydrogen bonds and alkyl bonds between Andro and the NF-kB (1NFI). Andro interacts with Asp-185 Asn-190 by conventional hydrogen bond, Ser-276 by carbon-hydrogen bond and also interacts with Pro-189, and Arg-278, by alkyl bond (Fig. 3C). COX-2 results illustrated that Andro possesses a high docking score of − 8.0 kcal/mol with 2 alkyl bonds at Leu-145 and val-538, conventional hydrogen bonds at Leu-224, His-226, and Val-228, whereas a single carbon-hydrogen bond was formed in Gly-533(Fig. 3F). The docking results suggests interactions between the ligand Andro with NF-kB and COX-2.

### The root mean square deviation (RMSD)

**Figure 4:**
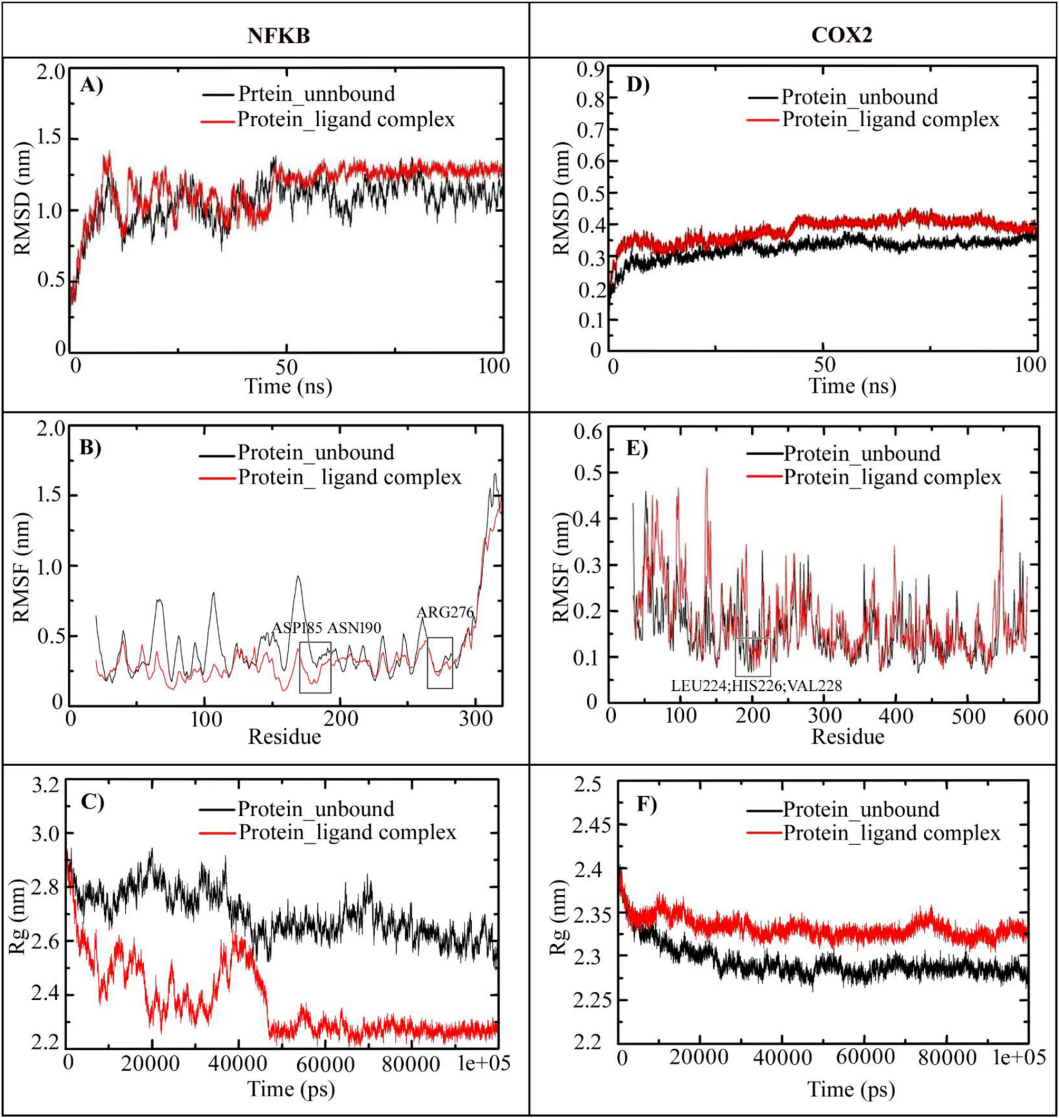
RMSD, RMSF, and Radius of gyration analysis for the native structure of 1NFI, 5IKT and its complex with Andro.

*1NFI and complex*: We performed MD simulation to analyse the conformational changes of the proteins 1NFI and 5IKT when interacting with the ligand Andro. The RMSD depicts the stability and structural convergence of the proteins before and after interacting with the ligand. In the derived RMSD for the protein 1NFI and protein–ligand complex 1NFI and Andro, the protein structure was flexible during the initial period of simulation from 0.4 to 1.25 nm (between 0 to 10.5 ns). The trajectory from 10.5 ns to 75 ns shows constant fluctuations; later from 75 ns protein attains equilibrium up to final MD with minimal fluctuations 0.25 nm closes at 1.10 nm. In the case of 1NFI and Andro, the complex structure was also flexible initially from 0.4 to 1.45 nm and continued with constant fluctuations till 47 ns. The complex trajectory was equilibrated and maintained its stability with minute fluctuations 0.06 till 100 ns, closing at 1.35 nm which is greater than the native 1NFI (Fig. 4A).

*5IKT and complex*: The obtained RMSD graph for the protein 5IKT and protein–ligand complex 5IKT and Andro, the protein structure was flexible during the initial period of simulation from 0.15 nm to 0.35 nm (between 0 to 35 ns). The trajectory from 35 ns protein attains equilibrium up to final MD with minimal fluctuations 0.1 nm closes at 0.35 nm. In the case of 5IKT and Andro, the complex structure was also flexible initially from 0.2 to 0.37 nm till 15 ns, and maintained its stability till 45 ns, with the least deviation of 0.1 nm at 45 ns reaching the fluctuation to 0.45 nm. The complex trajectory was equilibrated and continued with minute fluctuations 0.03 nm till 100 ns, merging with native 5IKT at 0.35 nm which is greater than the native 1NFI. From the RMSD analysis, it is clear that both the complexes of INF1 and 51KT with Andro are having low differences with native structures and attaining stability (Fig. 4D).

### The root mean square fluctuations (RMSF)

*1NFI and complex*: The RMSF analysis is used to evaluate the positional fluctuations of each residue of the protein. In this study, we have measured the fluctuations of residues in 1NFI native structure and protein–ligand complex structure during MD simulation and observed that the protein complex is having low fluctuations than that of the native structure. The residues from 286 to 320 were showing more fluctuations ranging from 0.75 to 1.5 nm indicating they are more solvent-exposed. The hydrogen bonds made by residues ASP185, ASN190, and ARG276 with ligand were showing significant troughs at 0.2 nm correlating with the docking studies as shown in Fig. 3, and these hydrogen bonds are restricting the fluctuations of the residues and affecting the overall fluctuations of the protein (Fig. 4B).

*5IKT and complex*: The residual mobility of the in the 5IKT in the absence and presence of the phytochemical Andro are plotted and analysed. It was observed that residues LEU224, HIS226 and VAL228 that involved in hydrogen bonding with the stable ligand showed lowest fluctuations at 0.08 nm. On contrary the residues from 125–150 showed highest peeks at 0.52 nm, indicating that the ligand is flexibilizing at these residues (Fig. 4E).

### Radius of gyration (Rg)

*INF1 and complex*: Rg is used estimate the compactness of the protein across the simulation that will help us to understand the folding properties of the protein. Lower the Rg higher the packing whereas higher Rg shows a floppy packing. Consistent Rg values through the time indicates that ligand holds the folding behaviour of the protein, whereas sudden fluctuations denote instability in the protein structure. In this study, the complex 1NFI is observed to have many fluctuations indicating its instability with its 51.5% random coils. Initially from (0–22 ns) the complex showed a notable low Rg value from 2.95 nm to 2.25 nm with consistent fluctuations. Since the ligand is bound to a predicted random coil region, making the protein undergo conformations. On the contrary from 48,000 ps (48 ns), the protein showed a sudden drop in the Rg and continued to be stable throughout closing at 0.29 nm (Fig. 4C).

*5IKT and complex*: As per the Rg graph plotted for the complex 5IKT with Andro against native protein, the Rg showed for the complex is slightly greater than the native with difference of 0.05 nm and remained the same throughout the simulation. Therefore, the Rg results suggest that ligand is insisting the slightest flexibility in the protein by closing at 2.325 nm whereas native is closing at 2.275 nm (Fig. 4F).

### Solvent accessible surface area (SASA)

**Figure 5:**
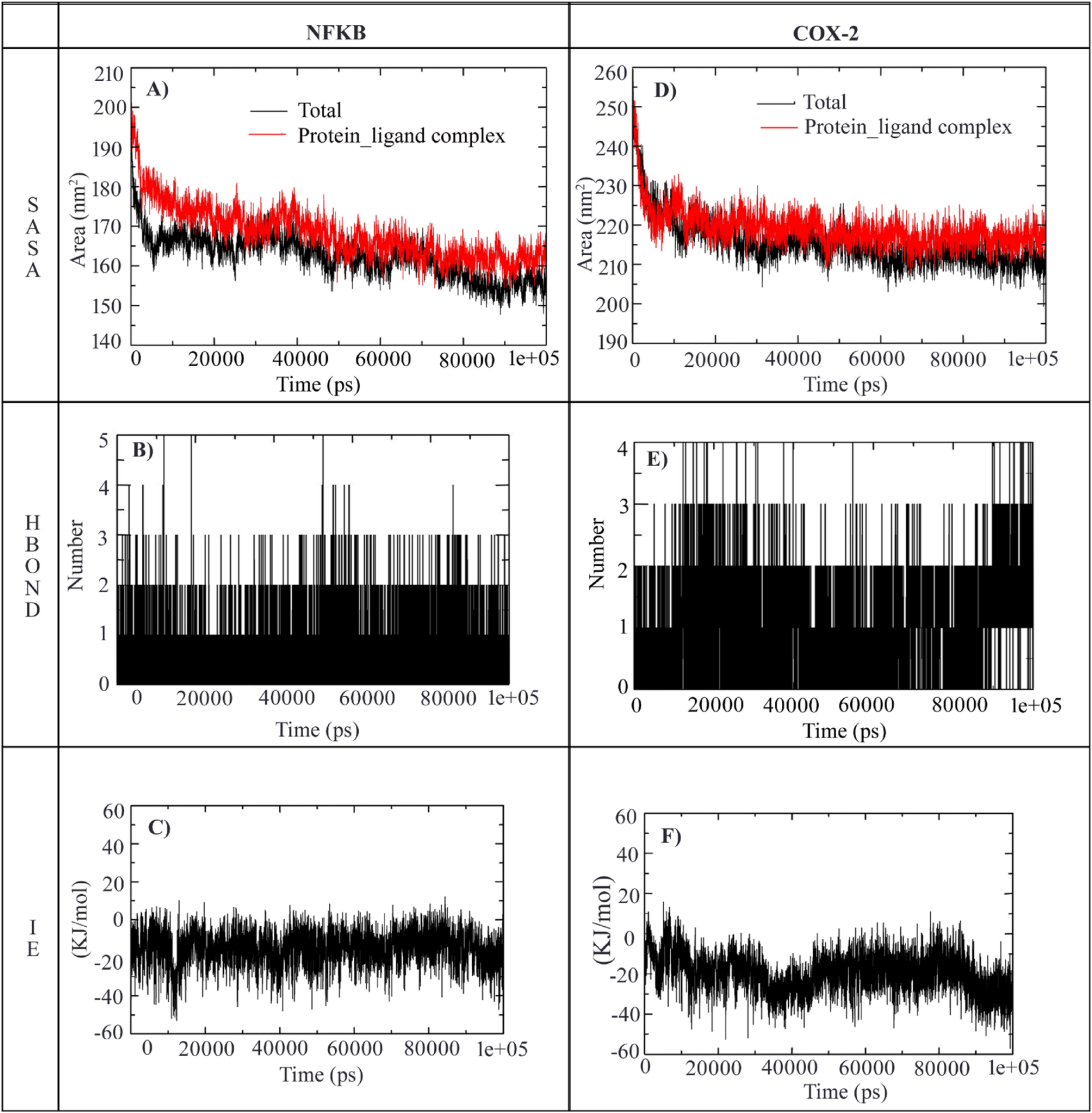
SASA, Hydrogen bonds, and IE analysis for the native structure of 1NFI, 5IKT and its complex with Andro.

*1NFI and complex*: In our study, we have calculated the SASA for the native protein of 1NFI and its complex with Andro and observed that the native starts from 190 nm^2^ and drops to 160 nm^2^ till 5000 ps (5 ns) and increases to 175 nm^2^ by 38,000 ps (38 ns) from there the accessibility to solvent gradually reduces and closes at 155 nm^2^ with an average of 162.55 nm^2^, whereas the complex showed little higher than native form starting at 200 nm^2^ closing at 165 nm^2^ with an average 168.27 nm^2^ reflecting that there is structural change hence surface residues are accessible to the solvent (Fig. 5A).

*5IKT and complex*: The SASA was checked with the native 5IKT in comparison with Andro complex to understand the protein surface area changes. In the beginning of the simulation the SASA of the native protein starts at 245 nm^2^ and has decreased up to 213 nm^2^ till 11,000 ps (11 ns) with an average of 215.46 nm^2^ whereas the complex starts at 250 nm^2^ and tends to decrease up to 215 nm^2^ till 5000 ps (5 ns) and again increased more than the native protein and continued to be the same all along with not much deviations and closes at 220 nm^2^ with an average of 219.09 nm^2^ (Fig. 5D).

### Hydrogen bond

*1NFI complex*: we have assessed the number of hydrogen bonds in the 1NFI complex versus time during the 100 ns MD simulation. The results suggested that there was maximum 5 hydrogen bonds in the complex out of which 3 hydrogen bonds were observed throughout the MD simulation (Fig. 5B).

*5IKT complex*: Where in the complex 5IKT with Andro there were maximum four hydrogen bonds observed, out of which 3 hydrogen bonds were seen throughout the simulation as depicted in the Fig. 5E. The observed hydrogen bonding parameters reflects that the ligand was bounded as effectively and tightly to both the complexes (i.e., 1NFI complex and 5IKT complex) (Fig. 5).

### Interaction energy (IE)

*1NFI* complex: IE was assessed along with the MM/PBSA analysis using GROMACS modules. In this analysis we have performed interaction potential energy (enthalpy) in kJ/mol, were average electrostatic energy of protein 1NFI – ligand complex was calculated as − 15.11 kJ/mol, and average van der Waals attractive energy was calculated as − 191.41 kJ/mol (Fig. 5C).

*5IKT complex*: The IE for the 5IKT complex has been evaluated by considering Coul-SR and van der Waals electrostatic LJ-SR, where the average enthalpy for Coul-SR: Protein-LIG was calculated as − 19.95 kJ/mol with − 190.37 kJ/mol van der Waal attractive energy (Fig. 5F).

### Andro suppressed the cervical cancer cell viability

**Figure 6:**
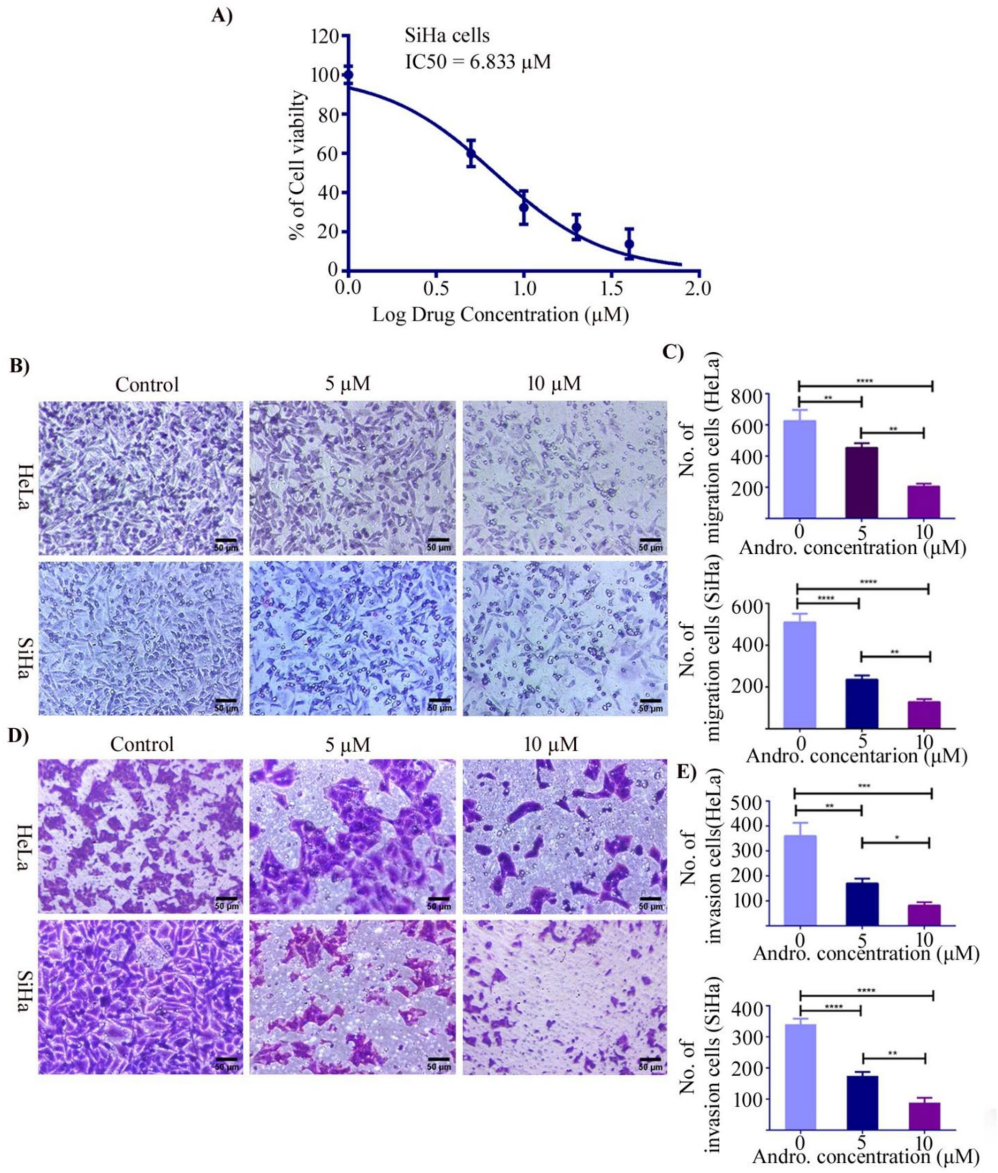
Effect of Andro on cell proliferation, migration and invasion of HeLa and SiHa cells assessed by MTT and transwell assay. (**A**) Cervical cancer cells, SiHa were cultured and incubated with various concentrations of Andro and the cell viability was measured by MTT assay. (**B**) Andro suppressed the cell migration in cervical cancer cell lines, HeLa and SiHa cells were treated with various concentrations of Andro (0, 5 and 10 µM) for 24 h. (**D**) Cell invasion was investigated in a matrigel-coated transwell cell culture chamber when HeLa and SiHa cells were subjected to various Andro concentrations (0, 5 and 10 µM). Cervical cancer cells potential of migration (**C**) and invasion (**E**) was measured by counting the number of cells using imagej software that migrated across or invaded the membrane’s underside. All the experiments were performed in triplicates and the data expressed as Mean ± SD. *P < 0.05, **P < 0.01, ***P < 0.001, ****P < 0.0001.

Earlier study from our lab has evaluated that Andro has an inhibitory impact on cervical HeLa cells at a concentration of 8.141 µM, whereas HEK cells were less sensitive, with an estimated IC_50_ value of 41.33 µM^48^. In the present study we have studied the effect of Andro on cervical cancer (SiHa cells) cell viability. The cells were treated with Andro for 24 h with various concentrations (0, 5, 10, 20, 40, 80 µM) and the cell viability was determined. After 24 h of Andro treatment, the IC_50_ value was estimated to be 6.833 µM (Fig. 6A). In contrast to our earlier investigations these results clearly reveal that SiHa cells are more sensitive to the effects of Andro therapy than Hela and HEK cells.

### Andro inhibits the cell motility of cervical cancer cells

The effects of Andro on the migration and invasion of CC cells (HeLa and SiHa) in-vitro were examined using the transwell chamber and the results are depicted in Fig. 6. The migration and invasiveness of cervical cells in response to chemo attractant (12% FBS) was investigated and the cells were treated with 0, 5 and 10 µM Andro (Fig. 6B,D). Andro had a significant inhibitory effect on cell migration at 5 and 10 µM concentrations in comparison to untreated control cells. As shown in Fig. 6C the migration upon Andro exposure on human cervical HeLa and SiHa cells was significantly suppressed in a dose dependent manner and inhibition was found to be 28%, 67% (HeLa) and 53% ,75% (SiHa) when cells were incubated with 5 and 10 µM Andro respectively. The invasiveness of HeLa and SiHa cells was determined by the matrigel coated membrane under the effect of FBS (12%) used as chemoattractant. Invasion assay results revealed that, in the absence of Andro, cervical cells moved from the upper chamber to the lower chamber (control group). However, Andro prevented cervical cells from penetrating the matrigel-coated filter. The inhibitory effect of Andro on cervical cells (HeLa and SiHa) at 5 µM was determined to be 53%, 30% and 77%, 49% at 10 µM, respectively (Fig. 6E). Together these results suggest that Andro significantly inhibited migration and invasion of cervical cancer cells in a dose dependent manner. Our research sheds light on the usage of natural compound, Andro as a potent alternative therapy for preventing metastatic cervical cancer.

### Andro attenuated NF-kB, COX-2 expression, and PTEN activation in cervical cancer

**Figure 7:**
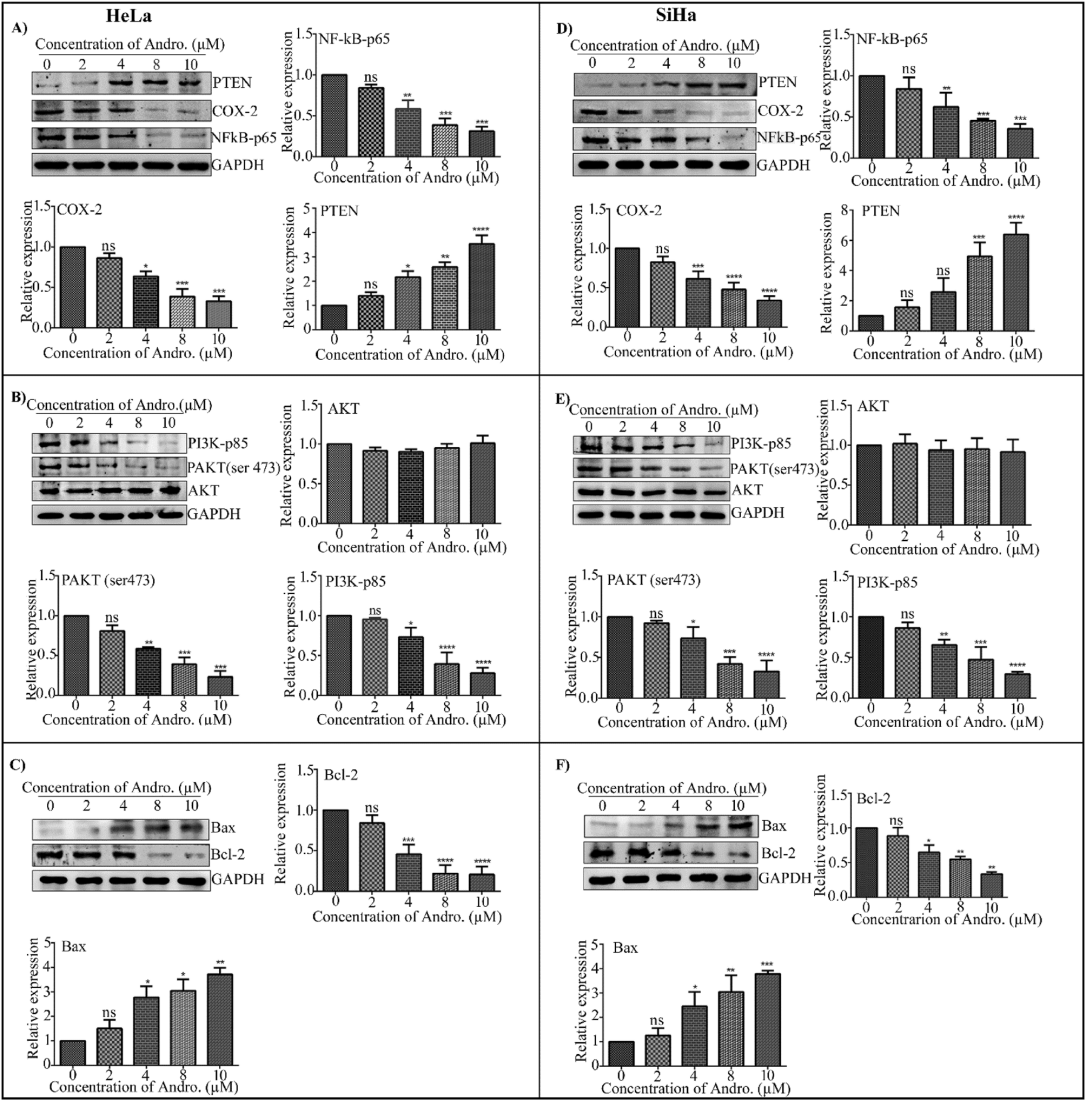
The effect of Andro on the NF-kB, PI3K, and apoptotic signalling pathways in cervical cancer cells. (**A**), (**D**) Expression of NF-kB, COX-2, and PTEN in cervical carcinoma, HeLa, and SiHa cells treated with Andro, i.e., 0, 2, 4, 8, and 10 µM cell lysates were subjected to western blot analysis with NF-kB, COX-2, and PTEN antibodies, followed by sequential re-probing against GAPDH. The bar graph depicts densitometric expression analysis of NF-KB, COX-2, and PTEN. (**B**), (**E**) Treatment with Andro i.e., 0, 2, 4, 8, and 10 µM cell lysates were subjected to western blot analysis with PI3K, p-AKT, and AKT antibodies in both cervical cancer cells HeLa and SiHa. GAPDH was used as a loading control. The bar graph depicts densitometric expression analysis of PI3K, p-AKT, and AKT. (**C**), (**F**) Cell death marker expression analysis: HeLa and SiHa cells were treated with 0, 2, 4, 8, and 10 µM Andro and subjected to western blot analysis with Bcl-2 and Bax. The bar graph depicts the densitometric expression analysis of Bcl-2 and Bax. All the blots were cut according to the molecular weight marker prior to hybridization with primary antibody for the corresponding protein. All the experiments were performed in triplicates and the data expressed as Mean ± SD. *P < 0.05, **P < 0.01, ***P < 0.001, ****P < 0.0001.

NF-kB governs the expression of various genes involved in a variety of processes, which include proliferation, migration, and cell death, all of which are crucial in the formation of tumor growth^19^. The NF-kB, regulates the expression of COX-2 and the overexpression of COX-2 supress the expression of PTEN^34,36^. PTEN inactivation is a key factor in the abnormal PI3K activation seen in many malignancies. Constitutive stimulation of the PI3K pathway promotes tumor growth^40^. Understanding the modulation of the NF-kB/COX-2, PTEN/PI3K pathways in cancer can provide insight into the malignant potential of tumor cells. Targeting these receptor molecules, such as NF-kB, COX-2, and PI3K, as well as PTEN, could be a novel approach for cervical cancer treatment. Further, we evaluated the inhibitory effect of Andro on NF-kB-p65, COX-2 and induction effect on PTEN expression in human CC HeLa and SiHa cells. Thus, to study the effect of Andro on the expression of both NF-kB-p65 and COX-2, HeLa and SiHa cells were treated with various concentrations of Andro (0, 2, 4, 8, 10 µM) and the levels of NF-kB-p65 and COX-2 was studied by immunoblotting. The immunoblot findings indicate significant immunoreactivity to NF-kB-p65 as well as COX-2 antibody, and Andro therapy inhibited NF-kB-p65 and COX-2 expression in both the cervical cells. We found a dosage-dependent reduction in the expression of NF-kB-p65, COX-2 and the highest evaluated concentrations of Andro (8 and 10 µM) was shown to be most effective against HeLa and SiHa cells (Fig. 7A,D). COX-2 expression levels gradually reduced as Andro concentration levels increased, whereas PTEN expression levels increased (Fig. 7A,D).

### Andro inhibits the PI3K p-85α/p-AKT signalling pathway in cervical cancer cells

ThePI3K is a promising therapeutic target, and inhibiting PI3K signalling is an effective treatment for a plethora of malignancies^38^. Recent studies have demonstrated that Andro suppresses the PI3K/p-AKT signalling pathway in breast cancer cells and human lung cancer^46,54^. We further investigated on whether Andro inhibited the PI3K/p-AKT signalling pathway in CC cells. By checking the expression levels of PI3K p-85α and p-AKT, the data revealed that Andro suppresses AKT phosphorylation and inhibited the expression levels of PI3 kinase regulatory subunit P85. The expression levels of p-AKT and PI3K downregulated in a dose dependent manner whereas no notable effect on AKT expression was seen (Fig. 7B,E). The findings suggest that Andro may inhibit cervical cancer progression by downregulating the PI3 kinase subunit P85α and thus suppressing AKT activation in cervical cancer cells.

### Andro induce apoptosis in cervical cancer cells

Apoptosis is a natural cell death mechanism that can be a promising target for anticancer therapy. We evaluated the inhibitory effect of Andro on apoptotic proteins Bcl-2 and Bax expression. The CC cells HeLa and SiHa were treated with 0–10 µM Andro for 24 h. The Andro treatment significantly increased the expression of pro-apoptotic protein Bax at 8 µM and 10 µM concentration and decreased the expression of anti-apoptotic marker Bcl-2 (Fig. 7C,F). The corresponding densitometric expression is shown in Fig. 7C,F, Andro increased the expression of the Bax protein in treated cells in a dose-dependent manner when compared to untreated cells, whereas the Bcl-2 protein was shown to decrease in a dose-dependent manner in CC cells.

### Andro inhibited tumor growth in cervical cancer xenograft model

**Figure 8:**
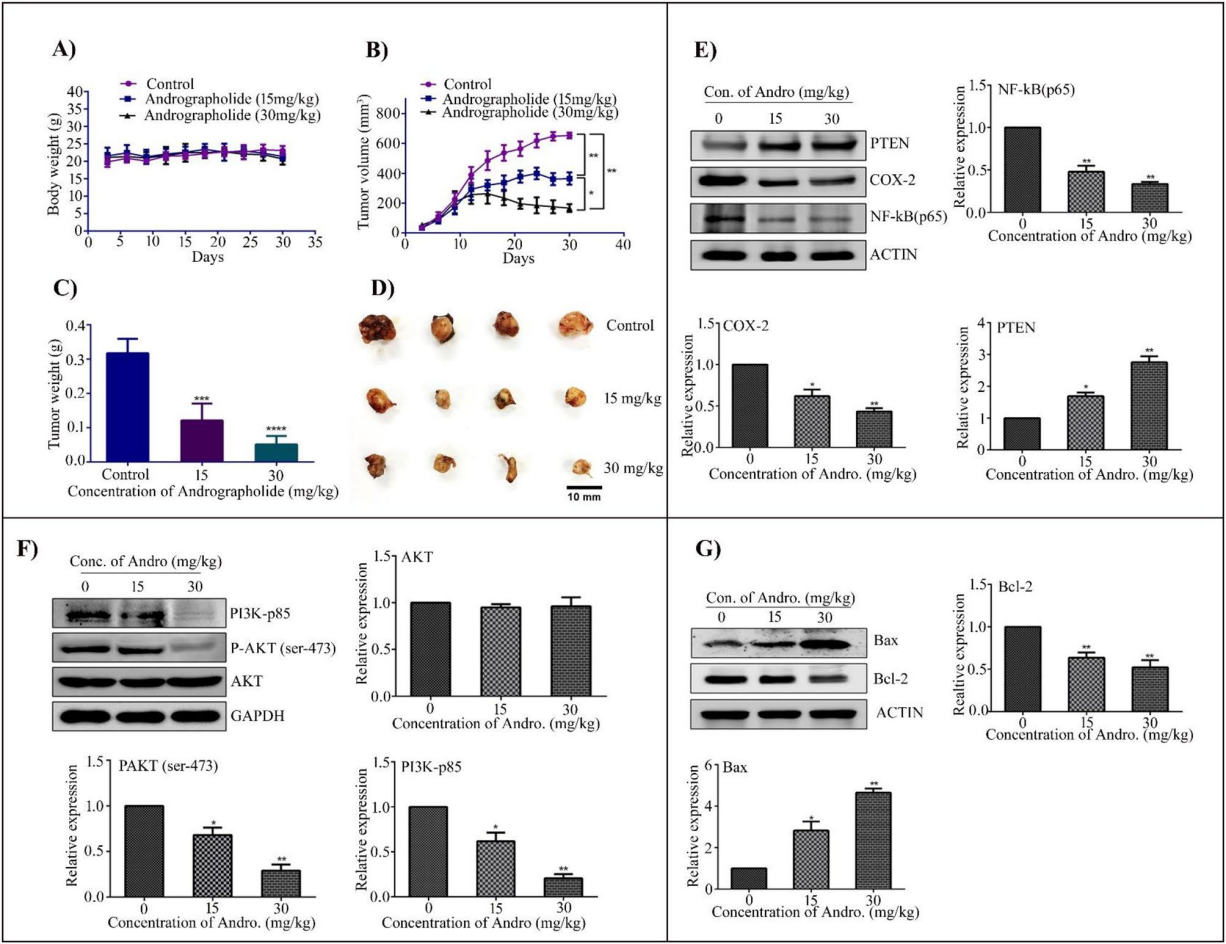
Inhibition of tumor growth by Andro in C57BL/6 mice Xenografted with HeLa cells. Tumor growth was recorded every 3 days by measuring its length and width with vernier caliper. Tumor size was calculated by tumor volume = Length × 2width/2. (**A**) Average body weight of mice, (**B**) Tumor volume, (**C**) Tumor weight, and (**D**) Tumor size. (**E**) Expression of NF-kB, COX-2, and PTEN in mice tumor tissues analysed by western blot analysis. Actin used as a loading control. The bar graph depicts densitometric expression analysis of NF-kB, COX-2, and PTEN. (**F**) Andro inhibits the PI3K/AKT pathway in mouse tumor tissues: western blot analysis and the bar graph showed that Andro significantly inhibited PI3K and AKT protein phosphorylation in mice treated with 15 mg/kg and 30 mg/kg in a dose dependent manner. (**G**) Andro induces apoptosis in mice tumor tissues: western blot analysis of anti-apoptotic protein Bcl-2 and pro-apoptotic protein Bax associated with apoptosis in mice tumor tissues following 15 mg/kg and 30 mg/kg Andro treatments. Actin is used as a loading control. All the blots were cut according to the molecular weight marker prior to hybridization with primary antibody for the corresponding protein. The quantitative data expressed as Mean ± SD. *P < 0.05, **P < 0.01, ***P < 0.001, ****P < 0.0001.

Based on the results of the in-vitro experiments, we established a CC xenograft model to study the anti-tumor activity of Andro in-vivo as explained earlier. Tumor volume and body weight were measured every three days, and tumors from all groups were collected at the end of the experiment. There was no significant change in the body weight of the mice in either of the treatment groups compared to the control group during the experimental period, indicating that the Andro treatment was not toxic to the experimental mice (Fig. 8A). As depicted in Fig. 8B, the tumor volume in control mice grows exponentially, but in mice treated with 15 mg/kg and 30 mg/kg of Andro, tumor growth was significantly reduced. Our findings demonstrated that, when compared to the control group, both the 15 mg/kg and 30 mg/kg Andro treatments significantly inhibited tumor volume and weight (Fig. 8B,C). The representative tumor picture in Fig. 8D demonstrated that the control mice have almost equivalent tumor size, whilst Andro treated mice have a reduction in tumor size.

### Andro inhibited NF-kB, COX-2, PI3K expression, and induces PTEN activation in mice tumor tissues

To further corroborate our in-vitro experimental data with the in-vivo studies, tumor samples were analysed by immnoblot analysis. Immuno blot analysis of the xenograft tumors revealed that Andro treatment increased the expression of PTEN while decreasing the expression of NF-kB and COX-2. The densitometric expression analysis of NF-kB, COX-2, and PTEN, as shown in Fig. 8E, revealed that treatment with 15 mg/kg and 30 mg/kg Andro significantly decreases the expression levels of NF-kB, COX-2, and significant increase in the expression of PTEN protein. In addition to, the cell survival pathway and apoptotic cell death markers were determined by immunoblot analysis of the xenograft tumor tissues. As shown in Fig. 8F, the immunoblot and densitometric results demonstrated that Andro significantly suppresses AKT phosphorylation and decreases PI3K-p85α expression levels, while AKT expression levels were not significantly altered when compared to the untreated control group.

The expression of Bcl-2 and Bax expression in mice tumor tissue samples showed that Andro treatment significantly induces apoptosis compared to the untreated control group. Andro decreased Bcl-2 expression while increasing Bax protein expression in tumor tissues from treatment group mice (Fig. 8G). The densitometric expression analysis of Bcl-2 and Bax protein demonstrated that Andro significantly increased the apoptosis in a dose dependent manner by up-regulating the expression of pro-apoptotic Bax protein.

**Figure 9:**
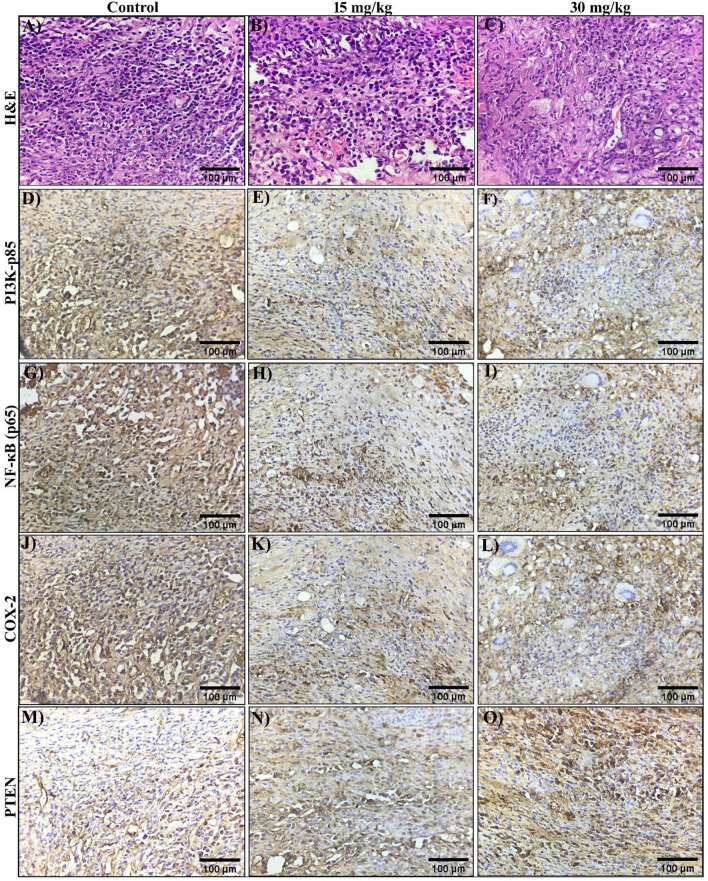
Histopathology and Immunohistochemistry expression analysis of mice tumor tissues. H&E staining of mouse tumor tissue sections (**A**–**C**), immunohistochemical expression analysis of PI3K (**D**–**F**), NF-kB (**G**–**I**), COX-2 (**J**–**L**), and PTEN (**M**–**O**) using specific primary antibodies, followed by diaminobenzidene (DAB) staining. All of the images were captured at a magnification of 40X.

Furthermore, tumor tissues from Andro-treated mice were examined by histopathology and immunohistochemistry to determine the precise mechanism involved in the regulation of the PI3K/AKT signalling pathway by activating the expression of PTEN via the suppression of NF-kB and COX-2. The histopathological results illustrated that in control tumor tissues, H & E-stained photomicrograph shows tumor tissue in cords as well as individually scattered showing moderate to highly pleomorphic cells with scant to moderate, clear to eosinophilic cytoplasm. Nuclei are deeply stained showing pleomorphism and increased nucleus to cytoplasmic ratio (Fig. 9A). Whereas, the mice treated with a lower dose of Andro, 15 mg/kg, shows scattered tumor cells as well as numerous blood vessels. Desmoplastic tissue replaces some tumor tissue. In mice treated with a higher dose of Andro (30 mg/kg), a large area of tumor tissue is replaced by desmoplastic tissue, as well as blood vessels and inflammatory cells, i.e., fibrocollagenous tissue-response to chemotherapy (Fig. 9B,C).

The IHC assay results showed that Andro inhibits the expression levels of PI3K, NF-kB, and COX-2 whereas increases the expression levels of PTEN as compared with control tissue. The detected PI3K and COX-2 expression in mice control tumor tissues is mostly diffuse and strong positive in the cytoplasm of tumor cells (Fig. 9D,J), whereas the NF-kB expression is diffuse, intense nuclear and cytoplasmic positive (Fig. 9G). The levels of PI3K, NF-kB, and COX-2 expression in tumor tissues of mice in the treatment group gradually decreased.

The expression levels of PI3K were seen in some areas of tumor tissue in treatment group-I (15 mg/kg), with diffuse, moderate cytoplasmic positivity (Fig. 9E), the expression levels of NF-kB are weak to moderate cytoplasmic and nuclear expression is seen in some areas (Fig. 9H), and the expression levels of COX-2 were seen in areas with scattered tumor cells show weak to moderate cytoplasmic expression (Fig. 9K). In contrast, treatment group II (30 mg/kg) showcased patchy weak cytoplasmic expression of NF-kB (Fig. 9I), diffuse mild positivity for PI3K expression (Fig. 9F), and a weak cytoplasmic expression of COX-2 (Fig. 9L). The expression levels of the tumor suppressor protein PTEN gradually increased in both treatment groups (Fig. 9N,O) compared to the control group (Fig. 9M). All together these findings suggest that Andro could effectively inhibit cervical cancer growth by down-regulating the expression of NF-kB and COX-2, and by activation of PTEN, inhibited the PI3K/AKT signalling pathway.

## Discussion

Cervical cancer is one of the most common types of cancer worldwide and the leading cause of death in women^1^. Despite the availability of several cancer-specific drugs, recent studies have focused on the anti-cancer properties of natural compounds for use in chemotherapy^54^. Tumor resistance to chemotherapy and molecular targeted therapy, whether intrinsic or acquired, can be diminished using multi-target therapy approaches^55^. Targeting multiple receptors that control cellular proliferation is the ideal course of action because tumors are heterogeneous and a single drug target approach may not be as effective. Recent studies have demonstrated that the transcription factor NF-kB is essential for the inflammatory response and the development of cancer by controlling the other inflammatory protein COX-2, which is crucial for the activation of the PI3K/AKT signalling pathway in cancer cells by suppressing the tumor suppressor protein PTEN^15,28,37^. Thus, targeting multiple receptor molecules such as NF-kB, COX-2, and PI3K, as well as PTEN, might be a novel strategy for cervical cancer management. It is preferable to identify novel plant-based compounds, with elevated anti-cancer activity with fewer side effects. Previous research demonstrates that Andro is one such compound induces apoptosis and regulates cell cycle arrest, but the mechanism governing this procedure still unknown.

In the current study, we used immunohistochemistry to compare the expression levels of NF-kB, COX-2, PI3K, and PTEN in cervical cancer tissue biopsy samples to normal cervical tissues, and the overexpression and downregulation of protein was correlated with cancer stage and metastasis. NF-kB-p65 expression was found strong nuclear positive and gradually increased with tumor stage (Stage II and III). In accordance with our findings, the recent studies found that NF-kB-p65 expression was strong nuclear positive in the majority of NSLCs (Non-small cell lung cancer) which is significantly correlated with tumor stage^56,57^. The canonical pathway member NF-kB can regulate COX-2 expression by binding to the COX-2 promoter region^26^. In the present study, we found elevated levels of COX-2 expression in the stromal region of cervical cancer tissue samples compared to normal cervix tissues and COX-2 expression levels gradually increased with tumor stage (Stage II and III). In accordance with present study, Nanyan jiang et al.^58^ found that increased COX-2 expression in CC tissues is positively related to cancer metastasis and stage. Earlier studies have shown that stromal cells in colorectal tissue express COX-2, and that COX-2 expression is strongly associated with NF-kB-p65 expression, suggesting that COX-2 induction may be mediated by activation of the canonical NF-kB pathway^59^. PI3K and PTEN are critical regulators of the PI3K/PTEN/AKT pathway, which regulate cell proliferation and survival. PTEN acts as a checkpoint for PI3K, which enhances the PI3K/AKT survival pathway. PTEN deficiency and high PI3K and mTOR expression are linked to a poor prognosis in triple negative breast cancer patients^60^. In our study the loss of PTEN immunoreactivity and upregulation of PI3K were noted in cervical cancer patients. Besides that, the current study found that loss of PTEN expression and overexpression of PI3K was associated with clinical stage of cervical cancer (stage II and III respectively). Qiufeng et al.^61^ and Loures et al.^62^ revealed that loss of PTEN expression plays an important role in the multiple steps of tumorigenesis and progression of cervical squamous cell carcinoma, which is consistence with our results. Real-time PCR results showed that mRNA expression levels were significantly elevated, which also correlates with IHC findings of overexpression of NF-kB, COX-2, PI3K, and loss expression of PTEN in cervical squamous tissues compared to non-tumor tissue.

Structure-based drug design allows for the virtual selection of possible drug molecules by assessing their binding affinity with their receptor proteins. In this study, we investigated the binding affinity of bioactive component, Andro from *A. paniculata* to macromolecular receptors, such as NF-kB, and COX-2. The binding affinity of the ligand to the proteins was determined, − 7.2 kcal/mol for NF-kB and − 8.0 kcal/mol COX-2. Jain et al.^63^ investigated the binding efficacy and stability of Andro and its analogue compounds as COX-2 inhibitors using molecular docking and molecular dynamics, and found that Andro has a binding affinity of − 7.95 kcal/mol to the COX-2 protein, which is in accordance with our result. The docking results indicated that the ligand, Andro could bind to the target proteins, NF-kB, and COX-2 more consistently in order to maintain the overall stability of the complex system. Andro used in the treatment of wide variety of disease conditions such as leprosy, ulcer, diabetes, influenza, skin diseases, colic, dysentery, dyspepsia and malaria^64,65^. The anticancer activity of Andro has been evidenced across several cancers and it inhibit cancer cell proliferation, arrest cell cycle and promotes apoptosis in human cancer cells^46,47,54,66,67^. Our previous study also suggested that Andro regulates the tumor cell proliferation, migration, induce apoptosis and arrest the cell cycle from G1 to S phase in human cervical HeLa cell lines^48^. These findings are consistent with recent studies indicating that Andro can inhibit colorectal carcinoma and breast cancer cell growth by arresting them in the G1-S phase^64^. Shi et al.^42^ found that Andro can impede colorectal carcinoma cell growth by arresting them in the G1-S phase, promoting the expression of p53, p21, and p16, which in turn suppressed the activity of Cdk2, Cdk4, and phosphorylation of retinoblastoma (Rb). To further demonstrate the effect of Andro on cell migration and invasion, we performed an MTT assay on tumor cell growth and found that treatment with Andro at inhibitory concentration 6.833 µM, significantly altered cell viability in cervical SiHa cells. In our previous study, the inhibitory effect of Andro was found to be 8.141 µM in cervical HeLa cells^48^. This dose range, above and below the IC_50_ values (5 and 10 µM), was employed in all following experiments to avoid the impact of cell growth on the observed parameters. Andro treatment inhibited both cervical HeLa and SiHa cell migration and invasion via in-vitro transwell analysis. Previous studies suggested that Andro could inhibit tumor cell motility, which is attributed to the tumor-endothelial interaction, migration, and invasive potential of cancer cells during metastasis^46,47,54^. Our findings indicated that Andro inhibits neoplastic progression and identified an extra passageway for its anti-cancer activity.

Numerous studies have shown that the NF-kB that regulates the expression of several genes such as COX-2, iNOS, MMPs, chemokines, and inflammatory cytokines, all of which involved in inflammatory and immune responses and promotes tumorigenesis^59,68^. In our previous study, we demonstrated that Andro has an anti-inflammatory effect by inhibiting iNOS expression and thus reducing NO production in CC HeLa cells^48^. Andro has an anti-inflammatory effect by inhibiting NF-kB binding to neutrophilic DNA, thereby reducing the expression of pro-inflammatory proteins like COX-2^66^. The current study demonstrated the response of human cervical cancer to Andro treatment and the results revealed that Andro significantly inhibited the growth of both HeLa and SiHa CC cells and induced apoptosis by downregulating NF-kB and COX-2 expression and activating PTEN. The immunoblot results showed that Andro reduced the expression of NF-kB-p65 and COX-2 in a dose-dependent manner, and the highest tested concentrations of Andro (8 and 10 µM) were found to be the most effective against HeLa and SiHa cells. Lee et al.^69^ revealed that Andro blocked LPS-induced iNOS and COX-2 in the RAW 264.7 cell line by inhibiting the activation of NF-kB and STAT3 through interference with SOCS1 and SOCS3 (suppressor of cytokine signalling). Loss or deletion of PTEN expression has been associated with constitutive expression of the PI3K/AKT pathway, and the abnormal activation of this pathway promotes tumor development and progression in majority of cancers^70,71^. Inhibiting constitutively active PI3K/AKT signalling can be a novel cancer treatment strategy. In accordance with these findings, we observed that Andro increased PTEN expression whereas reducing PI3K and pAKT expression levels in a dose-dependent manner, with no noticeable variation in total AKT levels.

Further the in-vivo studies have given us a better understanding of Andro's inhibitory effect on tumor size in CC xenograft model. We found that Andro treatments of 15 mg/kg and 30 mg/kg significantly reduced tumor size in xenograft mice. Andro inhibited the expression of PI3K, NF-kB, and COX-2 whereas increased the expression of PTEN when compared to control mice tumor tissue. Several in-vivo studies have shown that administration of Andro stimulated cytotoxicity and suppressed tumor formation in oral carcinoma xenografts^70^, and MDA-MB-231 tumor xenografts^46^. Andro significantly reduced tumor volume and tumor weights in a breast cancer xenograft model, and that the expression of COX-2 in transplanted tumors was significantly reduced in the treatment group as the dose of Andro increased^47^. Overall, our findings suggest that Andro treatment has the potential to inhibit tumor growth both in-vitro and in-vivo by targeting the multiple receptors that control cellular proliferation on human cervical cancer.

## Conclusion

The current study has investigated the NF-kB, COX-2, PI3K, and PTEN levels in cervical cancer patient samples at both the mRNA and protein levels. The expression of NF-kB, COX-2, and PI3K was found to gradually increase in CC tissues, whereas PTEN levels decreased significantly as the tumor progressed from stage II to stage III. The molecular docking, molecular dynamic, and simulation studies of NF-kB and COX-2 with the ligand Andro revealed encouraging and consistent results in docking and simulation studies. Our study deciphered the mechanism by which Andro induces apoptosis, blocks the tumor cell communication, and inhibits cervical cancer migration and invasion. We have shown for the first time that Andro has the potential to inhibit NF-kB and COX-2, as well as induce apoptosis in cervical cancer cells by impeding the PI3K/AKT signaling pathway. Andro could assist in retarding CC progression, increase the life expectancy of patients in advanced stages of cancer, and will also contribute to improved quality of life of affected women as compared to the women treated with chemo therapy. From the findings of the above study, administration of Andro could be an effective alternate safe plant-based compound to curtail and impede cervical cancer progression.

### Supplementary Information

Supplementary Information.

## Data Availability

Data supporting the findings of the study are available upon request from the corresponding author.

## Abbreviations

Andro: Andrographolide
CC: Cervical cancer
IHC: Immunohistochemistry
ROC curve: Receiver Operating Characteristics curve
NF-kB: Nuclear Factor kB
COX-2: Cyclooxygenase-2
PI3K: Phosphatidylinositol-3 kinase
PTEN: Phosphatase and tensin homolog
ARRIVE: Animal Research Reporting of In-Vivo Experiments
RMSD: Root mean square deviation
RMSF: Root mean square fluctuation
Hb: Hydrogen bond
Rg: Radius of gyration
IE: Interaction energy
SASA: Solvent accessible surface area
