# Supplementary material for: Inhibition of NF-kB and COX-2 by andrographolide regulates the progression of cervical cancer by promoting PTEN expression and suppressing PI3K/AKT signalling pathway
Source: Sci Rep. 2024 May 26;14:12020. doi: 10.1038/s41598-024-57304-7 (PMC11128455; doi:10.1038/s41598-024-57304-7)
Supplement: Supplementary file 1 — Supplementary Information. [file 41598_2024_57304_MOESM1_ESM.pdf]

Inhibition of NF-kB and COX-2 by Andrographolide regulates the progression of Cervical cancer by promoting PTEN expression and suppressing PI3K/AKT signalling pathway

Akbar Pasha[1], Kiran Kumar[2], SK Heena[3], I Arnold Emerson[2], Smita C. Pawar[1]*

[1]Department of Genetics & Biotechnology, University College of Science, Osmania University, Hyderabad 500007, Telangana, India

[2]Department of Bioinformatics, School of Biosciences and Technology, Vellore Institute of Technology, Vellore, Tamil Nadu 632014, India

[3]Department of Pathology, Osmania Medical College, Hyderabad, 500095 Telangana, India

*Corresponding author

Smita C. Pawar

Department of Genetics & Biotechnology, University College of Science, Osmania University, Hyderabad 500007, Telangana, India

Email address: smita.prof@gmail.com

Supplementary figure 1:

Original blots of cell lines (Hela, Siha) -figure 7 in the main text

(A, B, and C represent HeLa cell blots), (D, E and F represent SiHa cell blots)

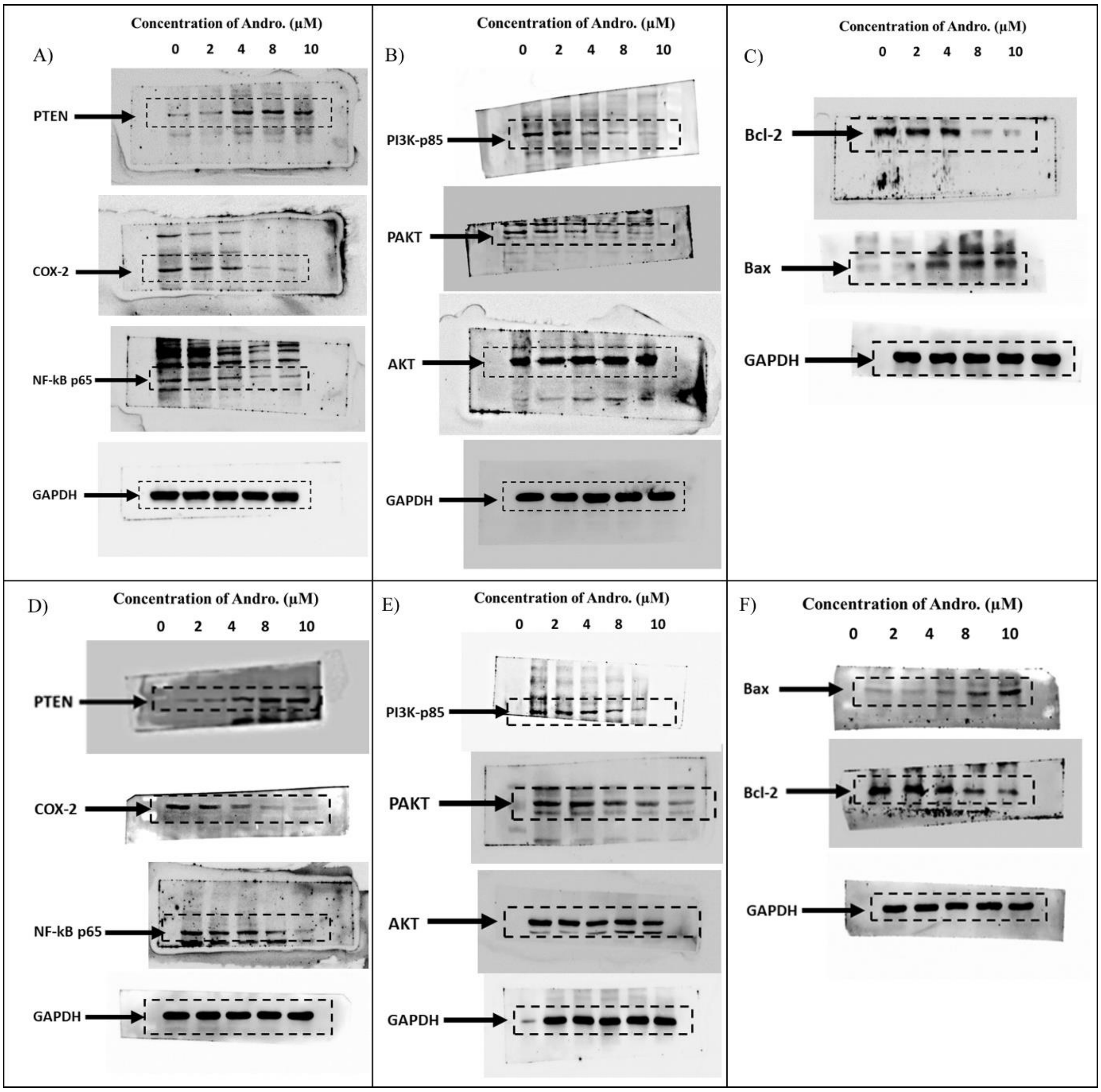

Figure 7: Primary set of blots

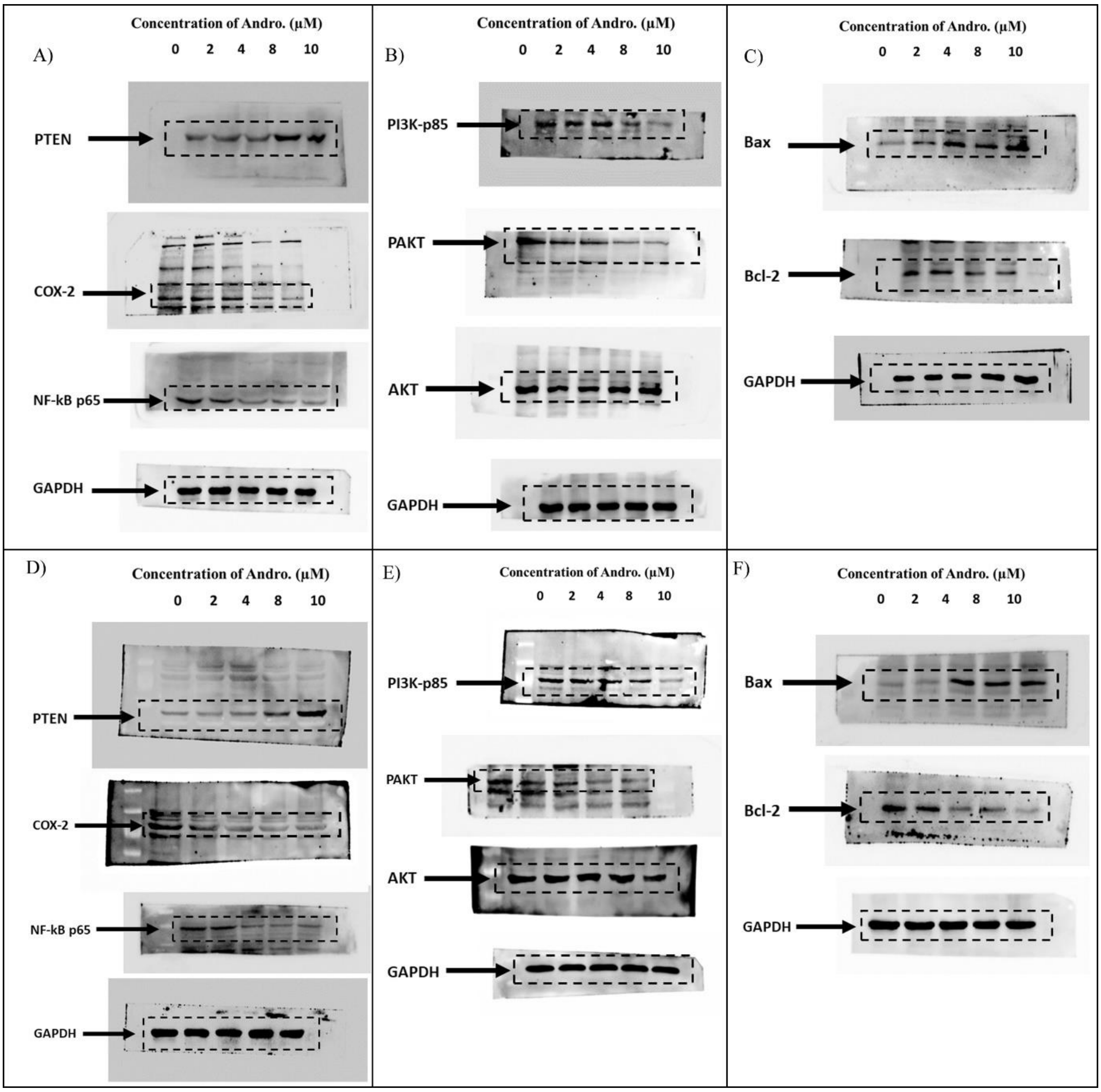

Figure 7: Replicate blots

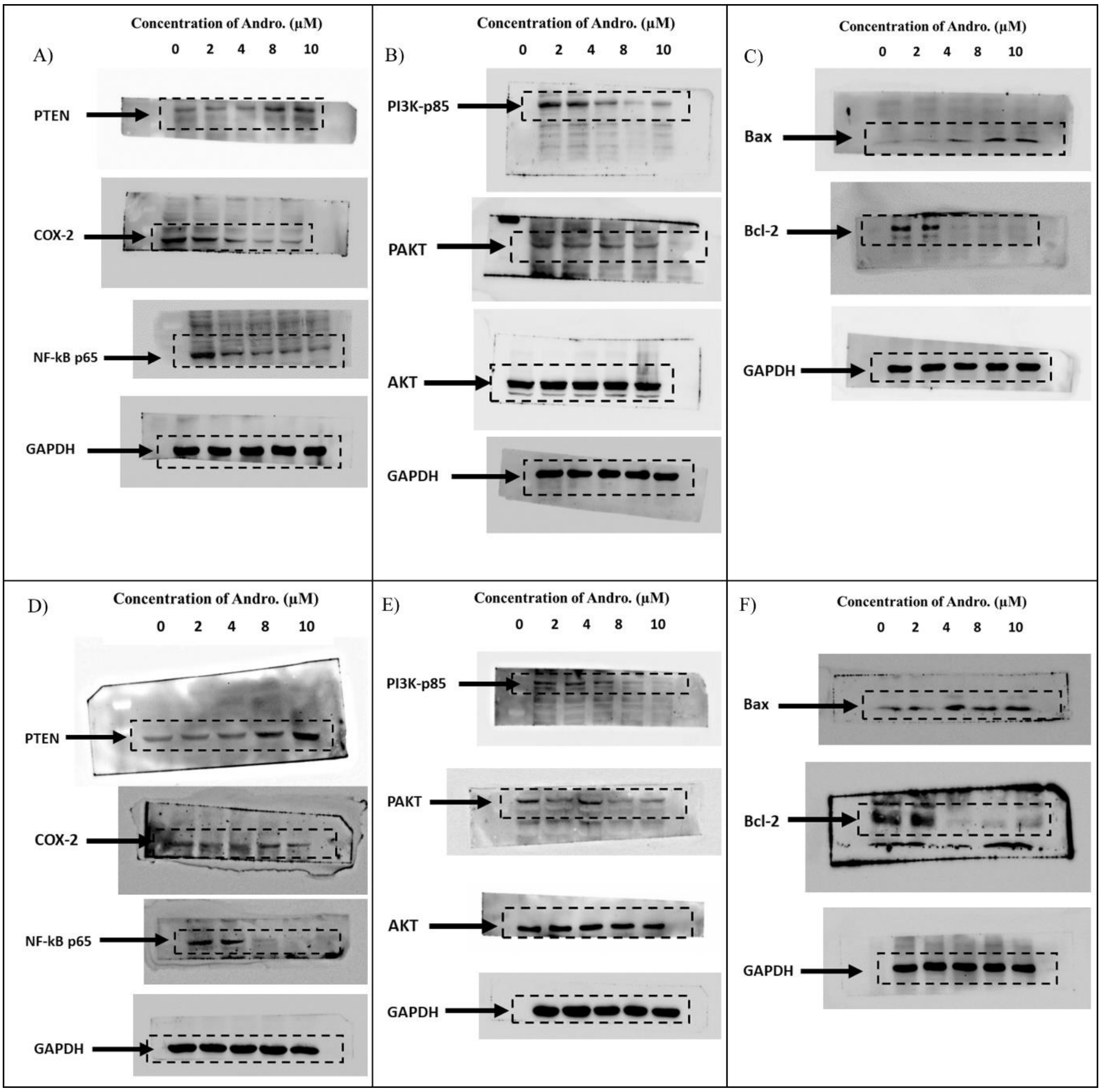

Figure 7: Triplicate blots

Figure 7: The effect of Andro on the NF-kB, PI3K, and apoptotic signalling pathways in cervical cancer cells. A), D) The expression of NF-kB, COX2, and PTEN in cervical cancer, HeLa, and SiHa cells treated with Andro at various concentrations (0-10 µM); B), E) Effect of Andro on PI3K/AKT pathway in cervical cancer cells (HeLa and SiHa); C), F) The expression of cell death markers in cervical cancer cells (HeLa and SiHa). All blots have been cut according to the molecular weight marker prior to hybridization with primary antibody for the corresponding protein.

Supplementary figure 2:

Original blots of mice tumor tissues-figure 8 in the main text

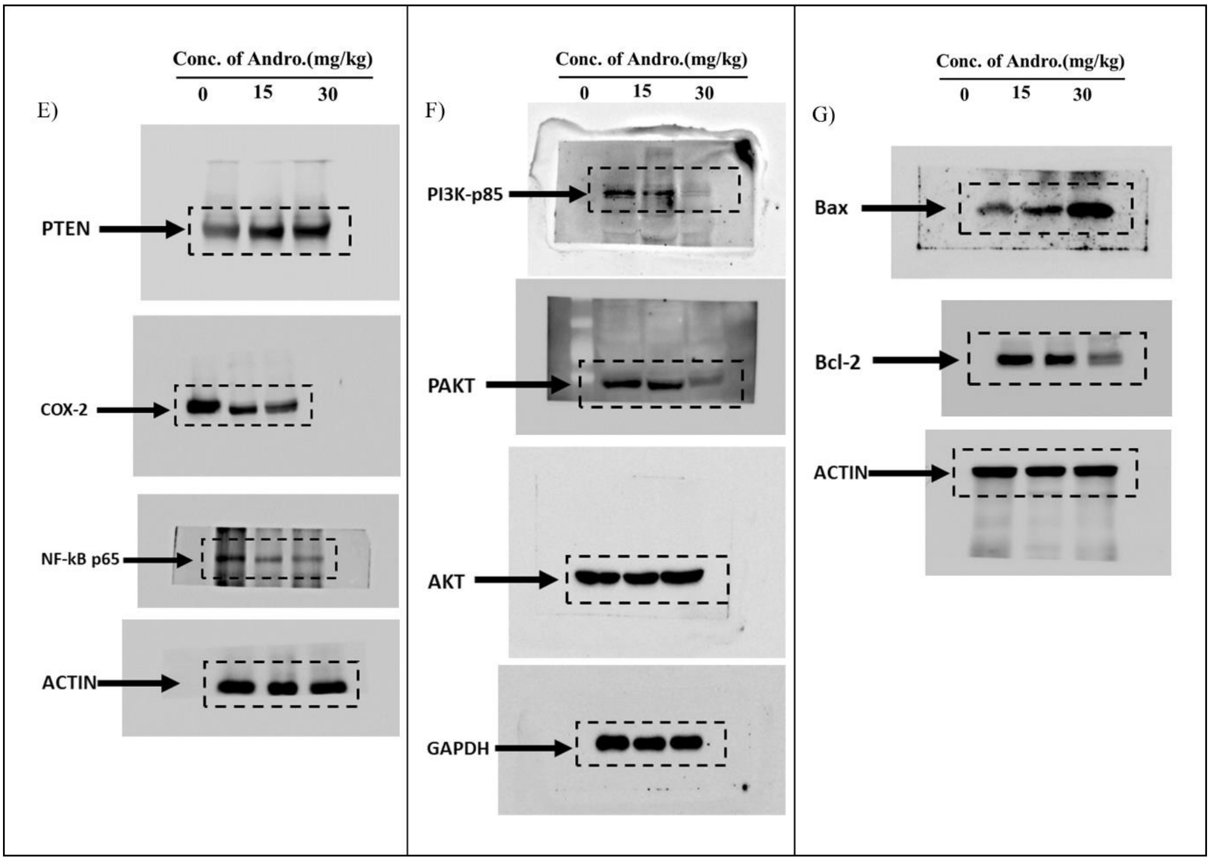

Figure 8: Primary set of blots

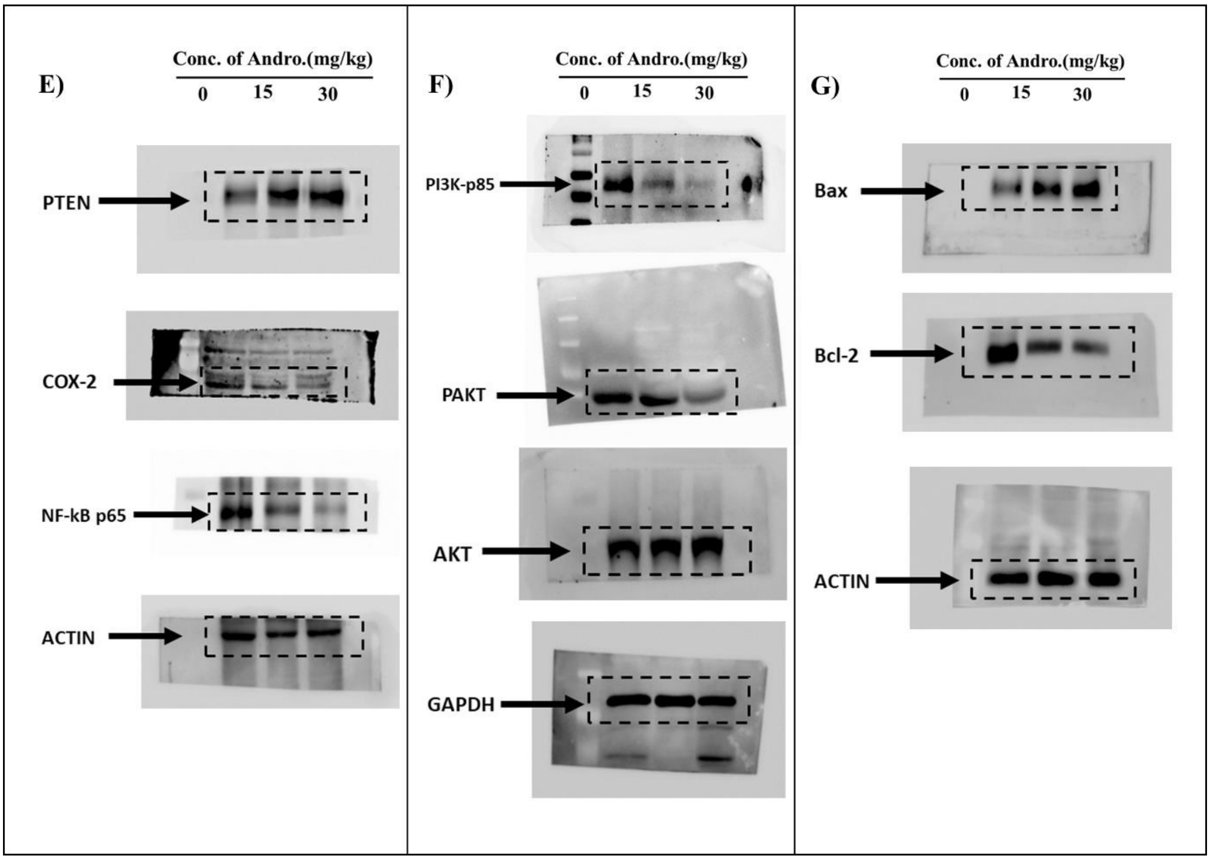

Figure 8: Duplicate blots

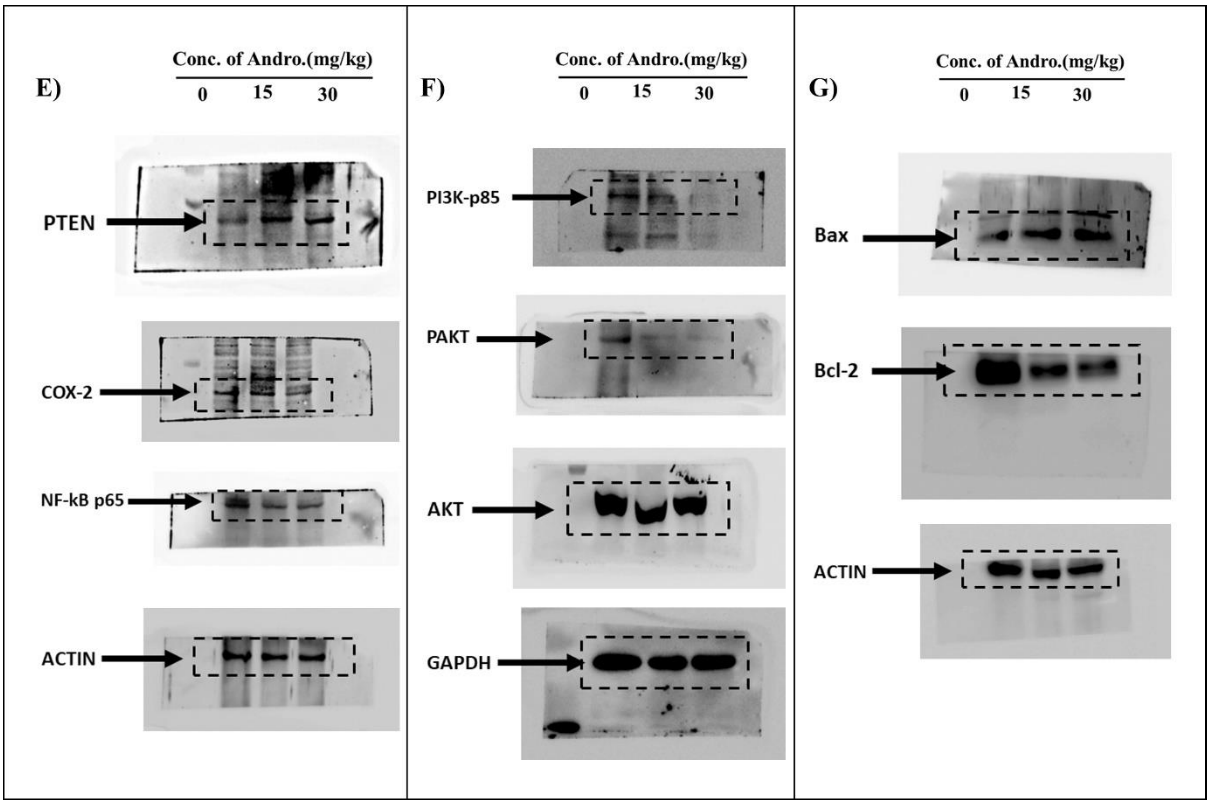

Figure 8: Triplicate blots

Figure 8: The effect of Andro on the NF-kB, PI3K, and apoptotic signalling pathways in mice tumor tissues. E) Expression of NF-kB, COX2, and PTEN in mice tumor treated with Andro at various concentrations (0, 15 mg/kg, 30mg/kg), F) The expression of PI3K/AKT pathway in mice tumor tissues G) The expression analysis of cell death markers in mice tumor tissues. All blots have been cut according to the molecular weight marker prior to hybridization with primary antibody for the corresponding protein.
